# New Sulfamethoxazole Derivatives as Selective Carbonic Anhydrase IX and XII Inhibitors: Design, Synthesis, Cytotoxic Activity and Molecular Modeling

**DOI:** 10.3390/ph15091134

**Published:** 2022-09-10

**Authors:** Mohamed A. Abdelgawad, Syed N. A. Bukhari, Arafa Musa, Mohammed Elmowafy, Mohammed H. Elkomy, AbdElAziz. A. Nayl, Ahmed H. El-Ghorab, Ibrahim Hotan Alsohaimi, Mohamed Sadek Abdel-Bakky, Ibrahim O. Althobaiti, Hamud A. Altaleb, Hany A. Omar, Ahmed H. Abdelazeem, Mohamed A. Zaki, Mohamed E. Shaker, Heba A. H. Elshemy

**Affiliations:** 1 Department of Pharmaceutical Chemistry, College of Pharmacy, Jouf University, Sakaka 72431, Saudi Arabia; 2 Department of Pharmacognosy, College of Pharmacy, Jouf University, Sakaka 72341, Saudi Arabia; 3 Department of Pharmaceutics, College of Pharmacy, Jouf University, Sakaka 72341, Saudi Arabia; 4 Department of Chemistry, College of Science, Jouf University, Sakaka 72341, Saudi Arabia; 5 Department of Pharmacology and Toxicology, College of Pharmacy, Qassim University, Buraydah 51452, Saudi Arabia; 6 Department of Chemistry, College of Science and Arts, Jouf University, Sakaka 72341, Saudi Arabia; 7 Department of Chemistry, Faculty of Science, Islamic University of Madinah, Madinah 41477, Saudi Arabia; 8 College of Pharmacy, University of Sharjah, Sharjah P.O. Box 27272, United Arab Emirates; 9 Department of Medicinal Chemistry, Faculty of Pharmacy, Beni-Suef University, Beni-Suef 62514, Egypt; 10 Pharmacy Department, College of Pharmacy, Riyadh Elm University, Riyadh 11681, Saudi Arabia; 11 Pharmacognosy Department, Faculty of Pharmacy, Beni-Suef University, Beni-Suef 62514, Egypt; 12 Department of Pharmacology, College of Pharmacy, Jouf University, Sakaka 72341, Saudi Arabia; 13 Pharmaceutical Organic Chemistry Department, Faculty of Pharmacy, Beni-Suef University, Beni-Suef 62514, Egypt

**Keywords:** carbonic anhydrase, anticancer, sulfamethoxazole, apoptosis, molecular docking

## Abstract

In this study new sulphamethoxazole derivatives (**S1**–**S4**, **S6**–**S12**, and **S14**–**S22**) were designed and synthesized and their structures were fully characterized and validated using NMR, mass, and IR spectroscopy, as well as elemental analyses. All new derivatives (**S1**–**S22**) were assayed against human carbonic anhydrase (hCAs IX and XII) for their inhibitory activities. hCAs IX and XII were chosen due to the fact that CAIX expression is recognized as a hypoxia marker with a poor prognosis in breast cancer. When compared to Dorzolamide HCl as a standard reference, derivatives **S2**, **S3**, **S8**, **S9**, and **S15** had the most effective inhibition with low IC_50_ values. The active compounds were further evaluated against hCAs I and II inhibitory activity and compounds **S8**, **S9** and **S15** showed the least inhibitory effect compared to the reference standard, acetazolamide, indicating that their effect in normal cells is the lowest. Cell viability tests for the selected compounds were carried out on MCF7 (normoxia and hypoxia) and on the normal breast cell line (MCF10a) with Staurosporine as a standard. The results showed that compound **S15** had a highly potent cytotoxic effect. Furthermore, cell cycle analysis results showed that compound **S15** triggered cell cycle arrest and apoptosis in G1/S of MCF7 cancer cells. Finally, molecular docking was performed to point out the possible explanation for the vital structural features and key-interactions exerted by our ligands with *h*CAs IX and XII that might share additional designs and highlight possible leads for a hopeful anticancer agent. Consequently, sulphamethoxazole Derivative **S15** could be the potential lead for emerging selective cytotoxic compounds directing *h* CAs IX and XII.

## 1. Introduction

Cancer is a disease that has a wide range of effects on humanity, starting with its negative influence on a patient’s physical and emotional health, and capacity to work and live a full life, and ending with its financial impact due to the high costs and prolonged course of cancer treatment [1]. In view of the seriousness of the disease and its harmful impact on human life, scientists have accelerated their endeavors to discover new and effective drugs [2].

In order to combat cancer, researchers need to find medications with the ability to target cancer cells without affecting healthy cells at a high pace of growth. As a result, identifying novel anticancer medicines relies heavily on the ability to identify specific and powerful anticancer targets. Human carbonic anhydrase enzymes (hCAs) are a recent target for anticancer drugs. These metalloenzymes, known as carbonic anhydrases (hCAs), are abundantly found in the human body and are involved in the synthesis of bicarbonate and proton from water and carbon dioxide (CO2) [3,4]. The hCAs catalyze various crucial reactions involved in pathological and physiological functions: for example, several metabolic processes (such as gluconeogenesis, ureagenesis and lipogenesis), respiration, bone resorption, pH and CO_2_, homeostasis, electrolyte excretion, and tumorigenicity [5].

A total of fifteen distinct hCA isoforms have been identified, and all but three of them are catalytically active, with the exclusion of CA-VIII, X, and XI, which lack the crucial histidine residues that are required for the coordination of the zinc ion [6]. Inhibition of hCAs has been shown to have substantial druggable therapeutic actions in many diseases, including cancer (hCAs-IX and XII), CNS-related disorders (*h*CAs-VII and XIV), glaucoma (hCAs-II, IV and XII), and edema (*h*CAs-II, IV and XIV) [7].

Overexpression of hCAs-IX and XII in malignancies has motivated medicinal chemists to design a novel target scaffold for finding new anticancer drugs to treat hypoxic cancers.

However, the design of high selective inhibitors for *h*CAs IX and XII, other than physiologically hCAs I and II isoforms, is a challenge for drug designers.

It was shown that the “tail style” of *h*CAs inhibitor development was the most developed, as well as the most effective [8].

Two arms witha broad range of chemical character, such as primary carboxylic and sulfamoyl function groups, are connected to a heterocyclic or aromatic ring comprising a zinc binding group (ZBG) by an adaptive and flexible linker. Sulphonamide or carboxylic groups of carbonic anhydrase inhibitors react with zinc, which is essential for hCAs activation.

Currently in Phase I/II clinical trials, a newly found hCAs inhibitor, SLC-0111, is highly selective for *h*CA IX over *h*CAs I and II isoforms in the treatment of solid hypoxic cancers [9,10].

Additional research has shown that, in combination with 3-O-acetylbetulin, SLC-0111 can increase radiation sensitivity, cytotoxicity and DNA damage while inhibiting cell motility. On the basis of this investigation, it has been indicated that a combination of SLC-0111 and other hypoxic tumour-targeting agents will have a promising therapeutic strategy [11].

**Figure 1:**
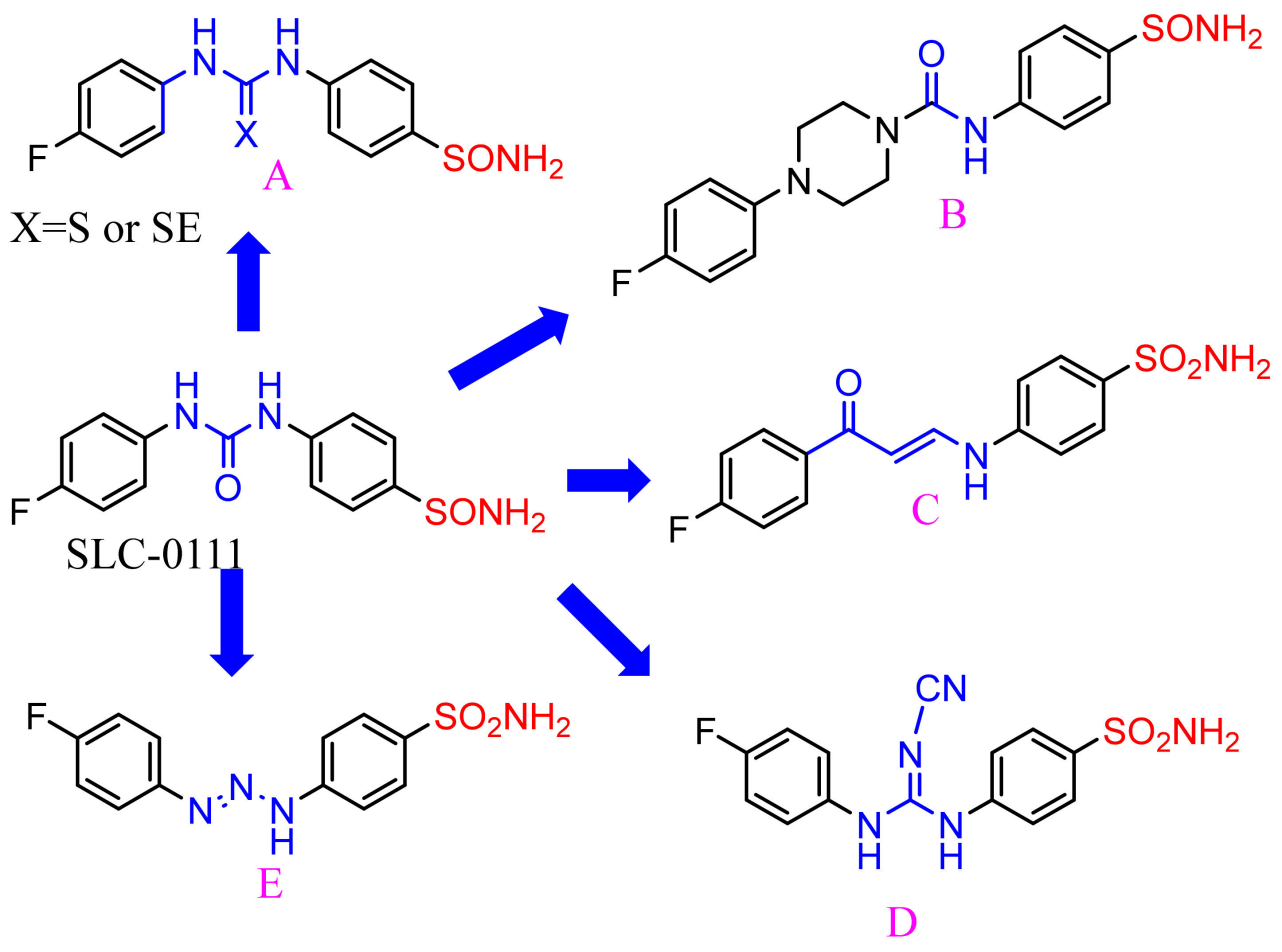
The main structural feature and similarity of reported *h* CA IX and XII inhibitors(**A**–**E**) with SLC-0111 (patent compound).

Bioisosteric substitution was also used by medicinal chemists to prepare new SLC-0111 derivatives. The SLC-0111 ureido bond has been substituted with selenoureido and thioureido (drug A), piperazinyl-ureido (drug B), enaminone (drug C), cyanoguanidine (drug D), and 1,3-triazene (drug E) linkers (Figure 1) [12,13,14,15,16].

These SLC-0111 bioisoteres, on the other hand, improved *h*CAs-IX and X11 inhibitory activity, but they did not show that they were more effective in blocking *h*CAs-IX than *h*CAs-I and *h*CAs-II. First, there is ZBG, which can be either sulpomyl or carboxylic or N-substituted sulfonamide. Then, there are flexible and variable linkers, and then the tail, which can be substituted with electron-withdrawing or electron-giving groups.

**Figure 2:**
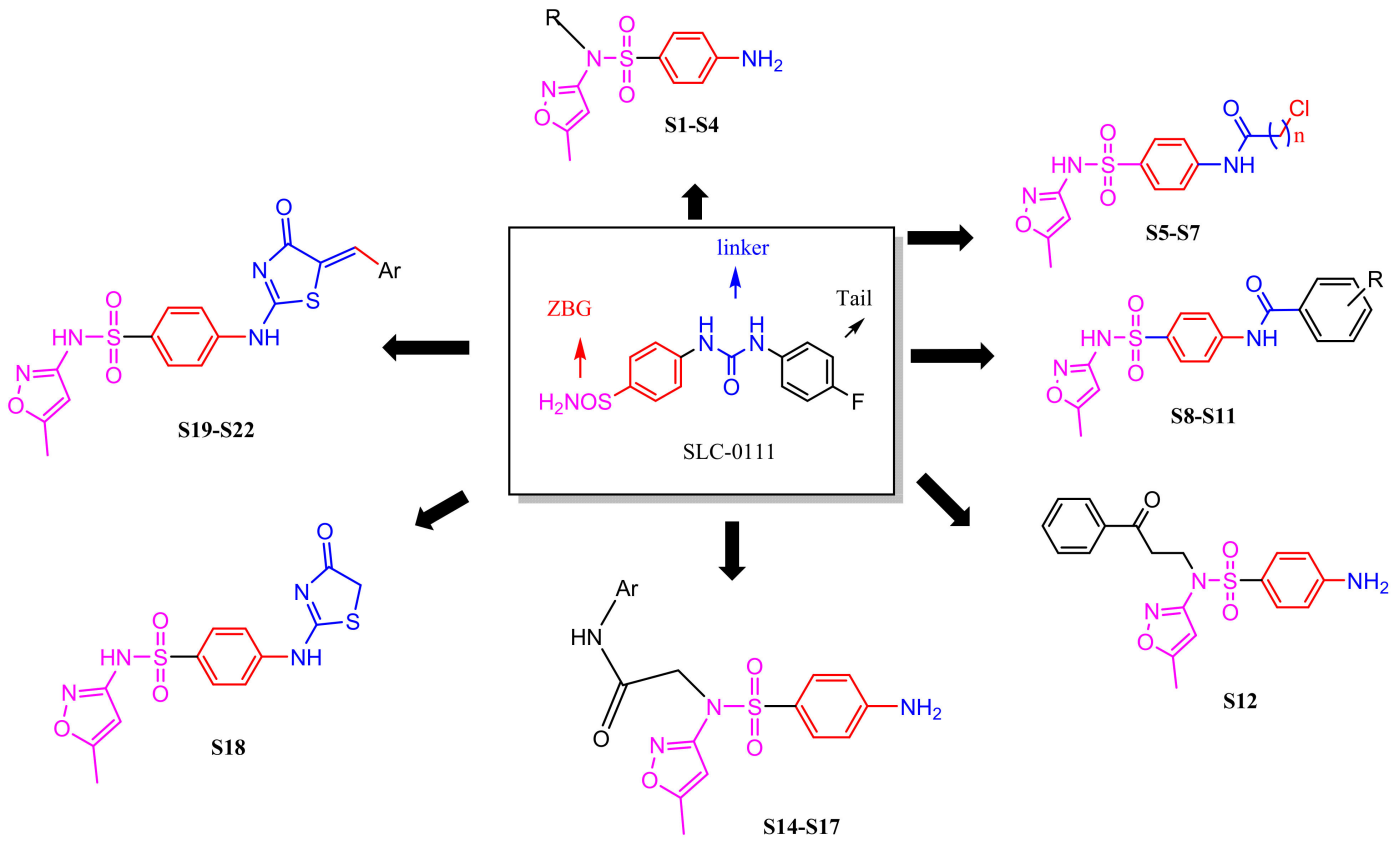
The main structural features and similarity of novel design h CA IX and XII inhibitors (**S1** to **S22**) with SLC-0111.

In this study, based on isoxazol-3-yl)benzenesulfonamide scaffold, we aim to develop and prepare new SLC-0111 derivatives with diverse functional moieties Figure 2.

The applied design approach depends on the substitution of the SLC-0111 ureido linker with substituted amino (**S1–S4**), chloroacylamido (**S5–S7**), substituted arylamido (**S8–S11**), benzoylethylamido (**S12**), substituted arylamido-acetamido (**S14–S17**), thiazolidinone-amino (**S18**) and substituted thiazolidinone-amino (**S19–S22**).

Moreover, a bioisosteric sulfonamide SLC-0111 with N-sunstiuted-sulfamoyl as ZBG, but the tail part of novel derivatives **S1** to **S22**.

Additionally, the 4-florophenyl group in the tail of SLC-0111 was replaced with substituted phenyl groups. All new compounds (**S1–S22**) were structurally verified, characterized, and pharmacologically assessed against *h*CAs I, II, IX, and XII, as well as tested for cytotoxicity against MCF7 and MCF10a cell lines. Additionally, the cell cycle and apoptosis of compound **S15** were analyzed.

The appropriate format for writing articles was followed: Abstract, Introduction, Methods, Results, Discussion, and Conclusions. In the current format, the writ-up is chaotic and boring to read. Hence, readers can lose interest.

The main objective of this study is to design and synthesize novel sulfamethoxazole derivatives that have the essential features of the reported compounds that have proven effective against carbonic anhydrase IX and XII. The novel sulfamethoxazole should be a potent and selective inhibitor for hCA IX and XII, which increase its secretion in the case of cancer and, at the same time, do not affect hCA 1 and II, which have vital and important physiological effects. 

As result, the new scaffold has proven to be effective against *h*CA IX and XII without harmful side effects resulting from action on physiologically important h CA 1 and II.

## 2. Results and Discussion

### 2.1. Chemistry

**Scheme 1:**
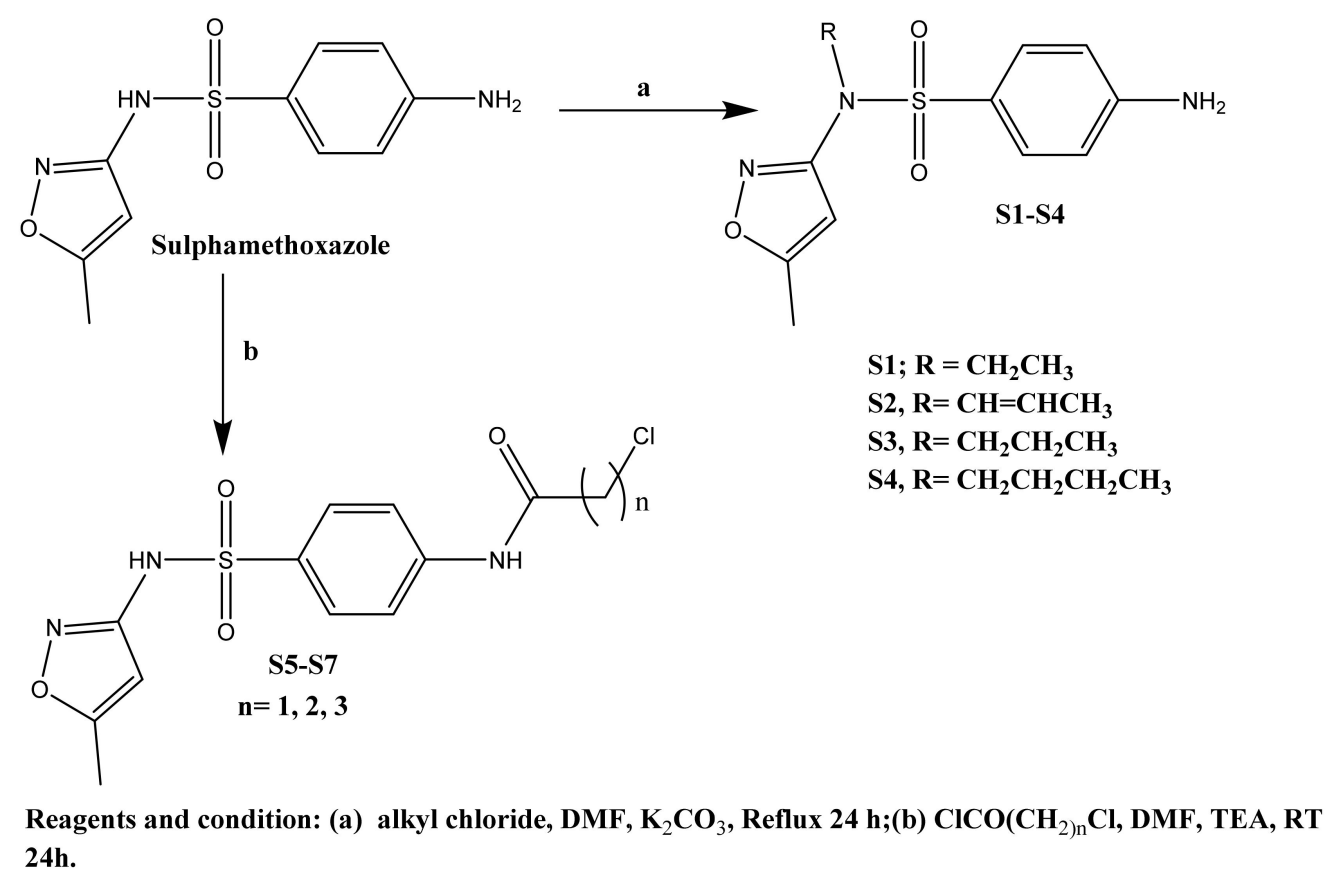
Synthesis of **S1** to **S7**.

**Scheme 2:**
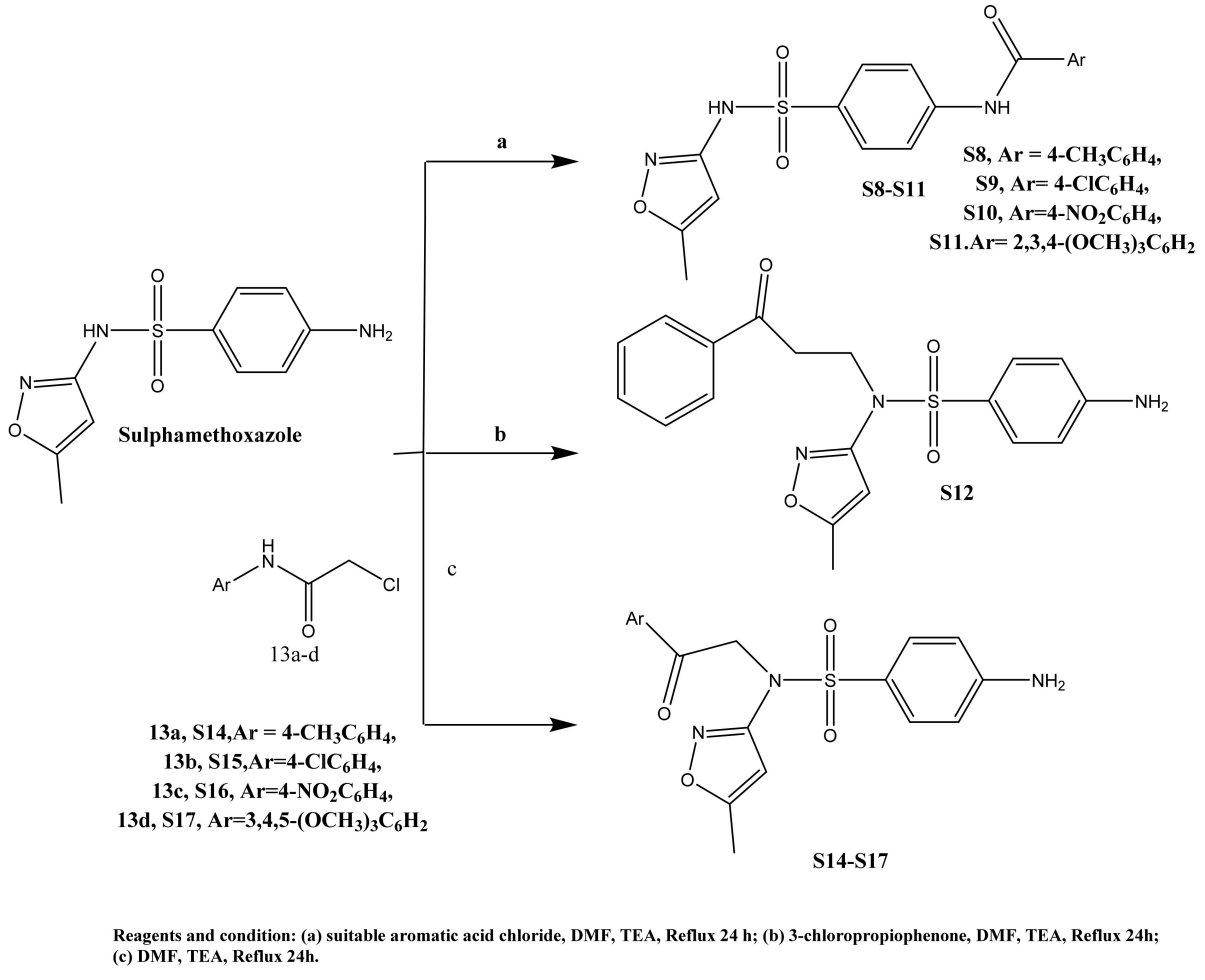
Synthetic pathway of **S8** to **S17**.

**Scheme 3:**
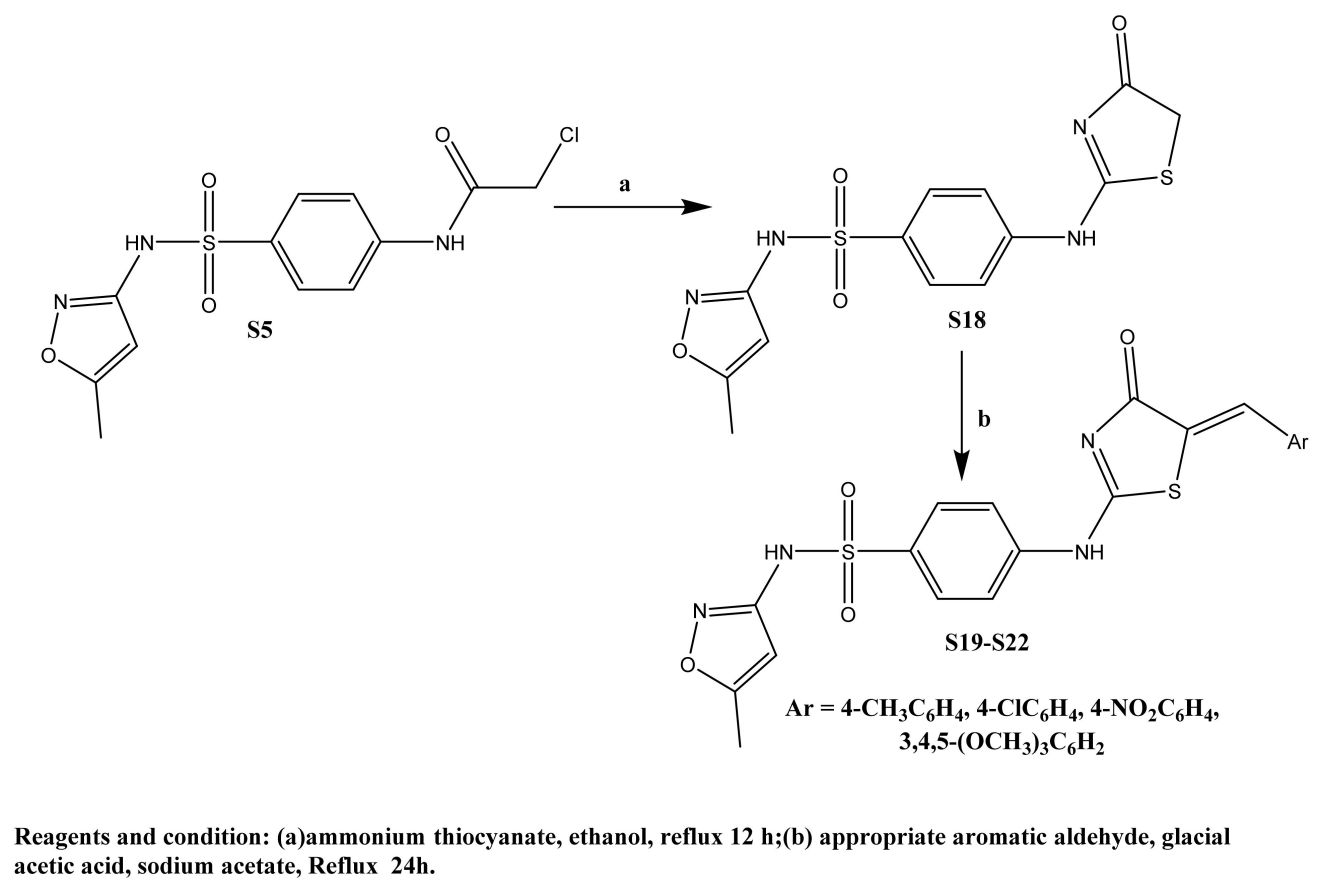
Synthesis of **S19** to **S22**.

The preparation of sulphamethexazole derivatives was performed according to the reaction orders demonstrated in (Scheme 1, Scheme 2 and Scheme 3).

Candidate derivatives **S1–S12** and **S14–S17** were prepared in an excellent yield via the nucleophilic substitution of sulphamethoxazole with different alkyl halide (for compounds **S1–S4**), acid chlorides (for compounds **S5** [17,18,19]**–S11**), 3-chloropropiophenone (for compound **S12**) or 2-chloroacetamide derivatives **S13a–d** [20,21,22,23] (for compounds **S14–S17**) in dry dimethylformamide in the presence of anhydrous potassium carbonate or triethylamine (Scheme 1 and Scheme 2).

The structure of novel prepared derivatives was well-characterized using NMR, IR and Mass spectroscopy, as well as elemental analyses.

The ^1^H NMR and ^13^CNMR of alkyl compounds **S1–S4** revealed the presence of aliphatic protons of different alkyl moieties at δ 1.16–5.85 ppm. Allyl group in compound **S2** appeared as a doublet, doublet of doublet, and multiple signals at *δ* 4.30, 5.20, and 5.75–5.85 ppm, respectively, in the ^1^HNMR spectrum.

The IR spectra of sulphamethexazole amide derivatives **S5–S11** showed absorption peaks at 1666–1687 cm^−1^ attributed to the carbonyl group. ^1^H NMR spectrum of **S6** displayed two triplets at *δ* 2.87 ppm and *δ* 3.88 ppm, each of them integrating two protons, which were accounted for CH_2_-CH_2_ of propionyl moiety. ^1^H-NMR of benzamide derivatives **S8–S11** displayed added signals at the aromatic region.

The structure of benzenesulfonamide derivative **S12** was confirmed by its spectral data. IR spectrum revealed a stretching band at 3331 cm*^−^*^1^ due to NH_2_ groups and a stretching band at 1765 cm*^−^*^1^ was ascribed to the carbonyl group. ^1^H NMR spectrum reported two triplets at *δ* 3.33 and 3.46 ppm, each equivalent to two protons with the same *J* value corresponding to CH_2_-CH_2_ protons. The presence of 3-oxo-3-phenylpropyl moiety was also confirmed by carbon NMR spectrum, which appeared at *δ* 37.72 (**C**H_2_CO), 39.71 (**C**H_2_-N) and 198.86 (CO).

In ^1^H NMR spectra of compounds **S14–S17**, additional protons for CH_2_ group and aryl moieties were detected at *δ* 4.47–4.59 and 6.63–8.23 ppm, respectively; in ^13^C NMR, the CH_2_ group appeared at a range of *δ* 50.66–50.90 ppm.

Cyclization of acetamide derivative **S5** with ammonium thiocyanate in ethanol under reflux conditions afforded thiazol-2-ylamino benzenesulfonamide derivative **S18** [24], followed by condensation with different aromatic aldehydes in glacial acetic acid to yield benzenesulfonamide derivatives **S19–S22** see Scheme 3. The structure of the new derivatives was assigned and established depending on their spectral data. The ^1^H NMR of compounds **S19–S22** confirmed the presence of benzylidene CH at *δ* 7.65–8.36 ppm.

### 2.2. Biological Evaluation

#### 2.2.1. hCA IX and XII Inhibiting Effect

**Figure 3:**
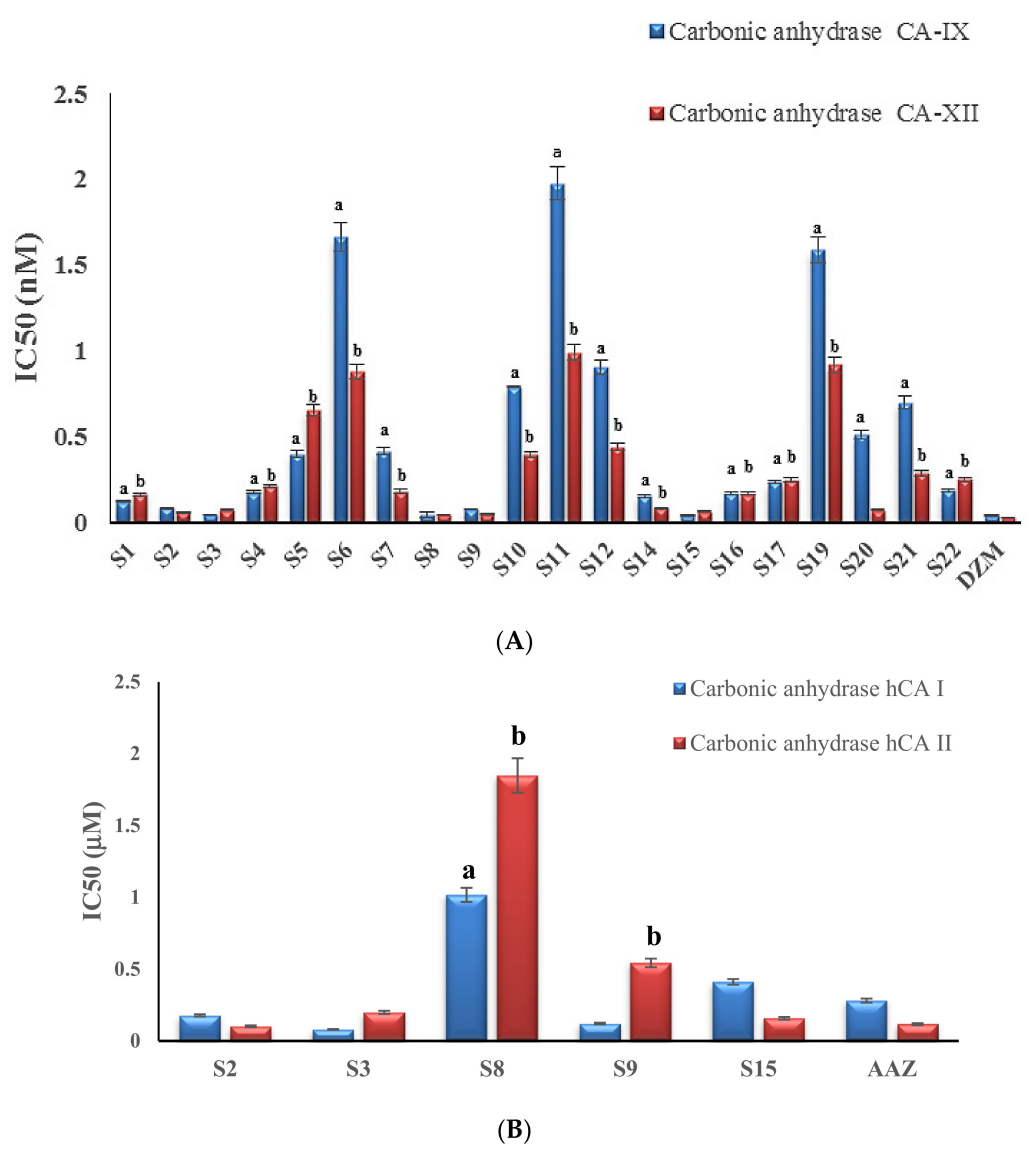
(**A**) Human CAIX, XII inhibitory effect of **S1–S22** compounds using Dorzolamide HCL as reference standard. ^a^ and ^b^ consider statistically significant at *p* < 0.05 if compared to DZM for CA-IX and CA-XII, respectively. (**B**) Human CAI, II inhibitory effect of **S2**, **S2**, **S8**, **S9** and **S15** compounds. Data represent mean ± SEM after three independent experiments. ^a^ and ^b^ consider statistically significant at *p* < 0.05 if compared to AAZ for hCAI and hCAII, respectively.

In the current experiment, *h*CA inhibiting effect was performed on 22 compounds and Dorzolamide HCL (DZM) was used as a reference standard. Out of these compounds, **S2**, **S3**, **S8**, **S9** and **S15** showed potent *h*CA IX inhibiting effect with IC_50_ of 0.083 ± 0.004, 0.042 ± 0.002, 0.042 ± 0.002, 0.074 ± 0.004 and 0.037 ± 0.002, respectively, compared to DZM (IC_50_ of 0.036 ± 0.002). Similarly, **S2**, **S3**, **S8**, **S9** and **S15** showed strong *h*CA XII inhibiting action with IC_50_ of 0.056 ± 0.003, 0.07 ± 0.003, 0.04 ± 0.002, 0.047 ± 0.002 and 0.061 ± 0.003, respectively, compared to DZM with IC_50_ of 0.024 ± 0.001 (Table 1 and Figure 3A,B).

#### 2.2.2. hCA I and II Inhibiting Effect

In the current experiment, compounds **S2**, **S3**, **S8**, **S9** and **S15** were selected for *h*CA I and II inhibiting activity based on the results of screening analysis on CA IX and IX inhibiting effect. Remarkably, compounds **S2**, **S3**, **S8**, **S9** and **S15** revealed a potent inhibitory effect on *h*CA IX with IC_50_ of 0.177 ± 0.008, 0.08 ± 0.003, 1.017 ± 0.049, 0.121 ± 0.006 and 0.411 ± 0.02, respectively, compared to the reference standard, Acetazolamide (AAZ), with IC_50_ of 0.281 ± 0.014 (Table 2 and Figure 3). Likewise, compounds **S2**, **S3**, **S8**, **S9** and **S15** revealed potent inhibitory action on CA XII with IC_50_ of 0.102 ± 0.006, 0.199 ± 0.011, 1.848 ± 0.12, 0.543 ± 0.03 and 0.158 ± 0.009, respectively, compared to AAZ with IC_50_ of 0.117 ± 0.007 (Table 2 and Figure 3). Notably, compounds **S8**, **S9** and **S15** showed the least inhibitory effect compared to compound **S2** and **S3** and the reference standard, AAZ, indicating that their effect in normal cells is the lowest.

#### 2.2.3. Cytotoxic Activity against MCF7 and MCF10a Cell Lines

**Figure 4:**
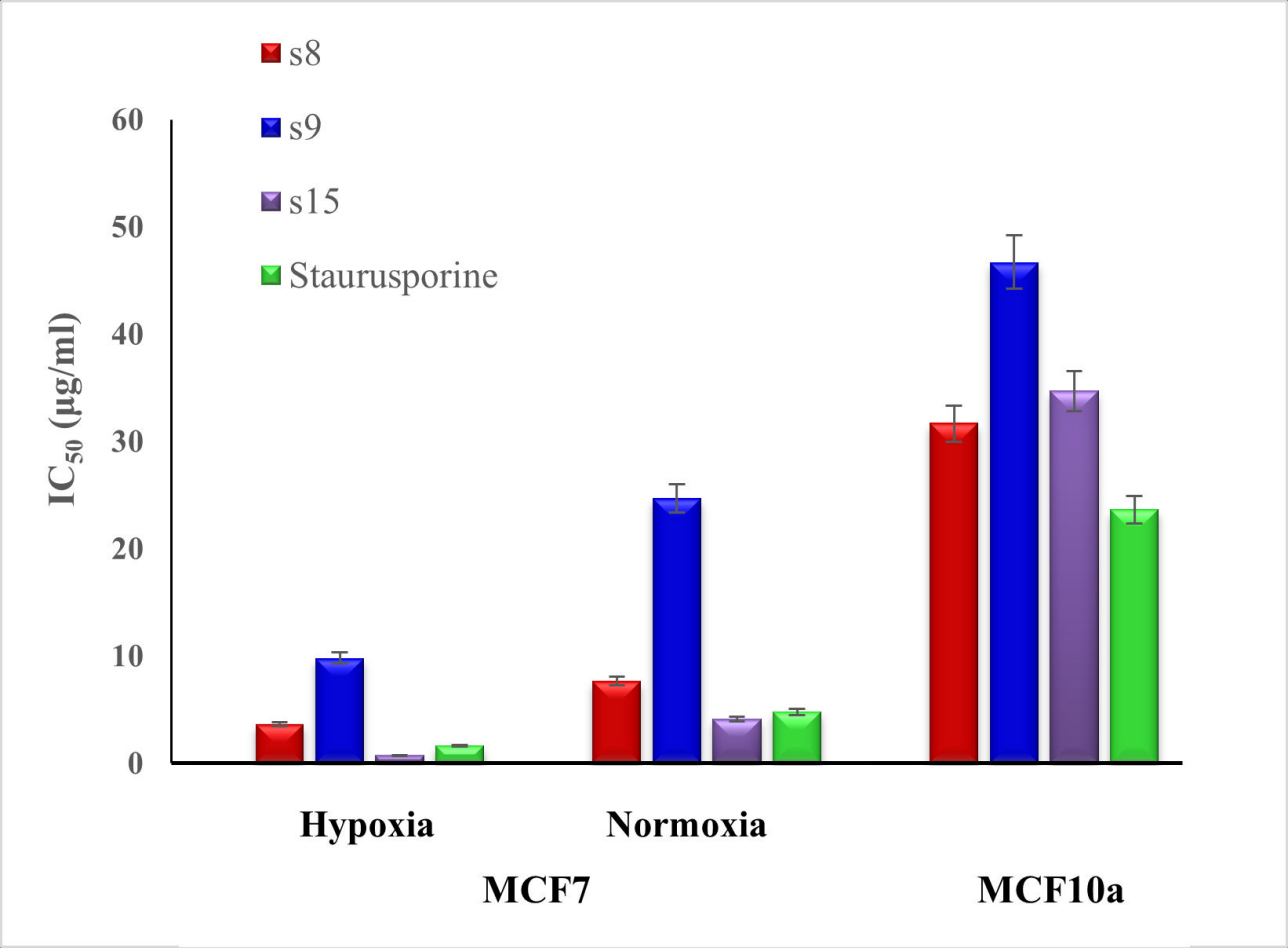
Effect of **S8**, **S9** and **S15** compounds on cell viability of MCF7 (normoxia and hypoxia) and MCF10a cells using Staurosporine as reference standard. Data expressed as mean ± SEM.

The in vitro cytotoxic activity for **S8**, **S9** and **S15** compounds was estimated on MCF7 and MCF10a cell lines. Staurosporine was used as a reference standard in the current experiment. The concentration of the derivative that causes the 50% inhibition of cell survivability (IC_50_) was calculated. Table 3 and Figure 4 show the IC_50_ of the synthesized compounds compared to Staurosporine. Based on the results of the CA assay, **S8**, **S9** and **S15** compounds were selected for the cytotoxicity study. In the current study, the results displayed that compounds **S15** and **S8** had strong cytotoxic activity on MCF7 in the hypoxic conditions with IC_50_ of 0.73 ± 0.04 and 3.64 ± 0.2 μg/mL, respectively, compared to Staurosporine (IC_50_ of 1.63 ± 0.09 μg/mL). Similarly, compounds **S15** and **S8** had strong cytotoxic activity on MCF7 in the normoxic condition with IC_50_ of 4.15 ± 0.2 and 7.68 ± 0.41 μg/mL, respectively, compared to Staurosporine (IC_50_ of 4.78 ± 0.26 μg/mL). Instead, compounds **S8** and **S15** showed potent cytotoxic activity on MCF710a recording IC_50_ of 31.65 ± 1.71 and 34.71 ± 1.87 μg/mL, respectively, compared to Staurosporine (IC_50_ of 23.65 ± 1.27μg/mL). Notably, compound **S9** showed moderate IC_50_ activity in hypoxic and normoxic conditions on MCF7 and IC_50_ on MCF10a, counting 0.73 ± 0.04, 4.15 ± 0.22 and 34.71 ± 1.87, respectively (Table 3 and Figure 4).

#### 2.2.4. Cell Cycle Analysis and Apoptosis of Compound **S15**

**Figure 5:**
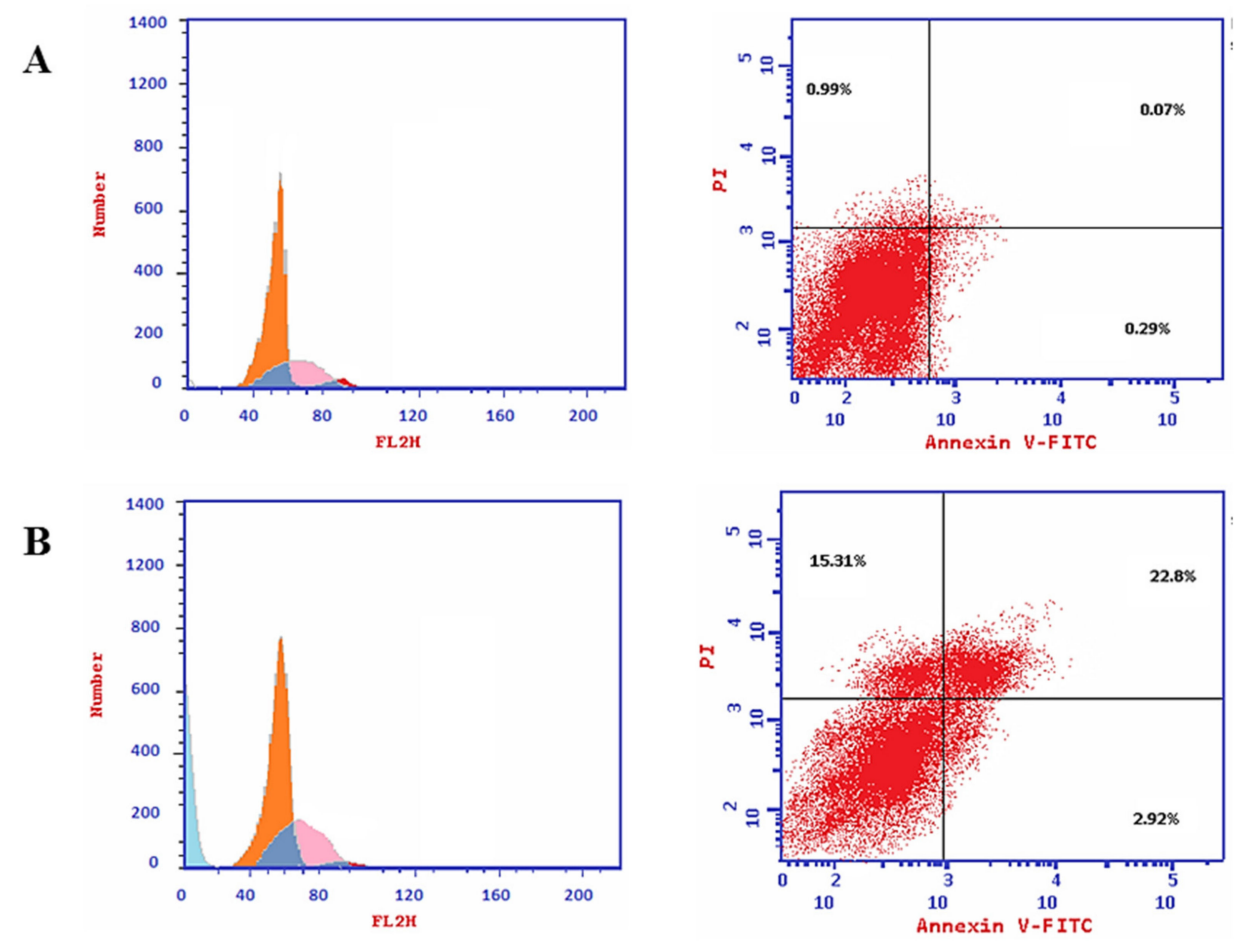

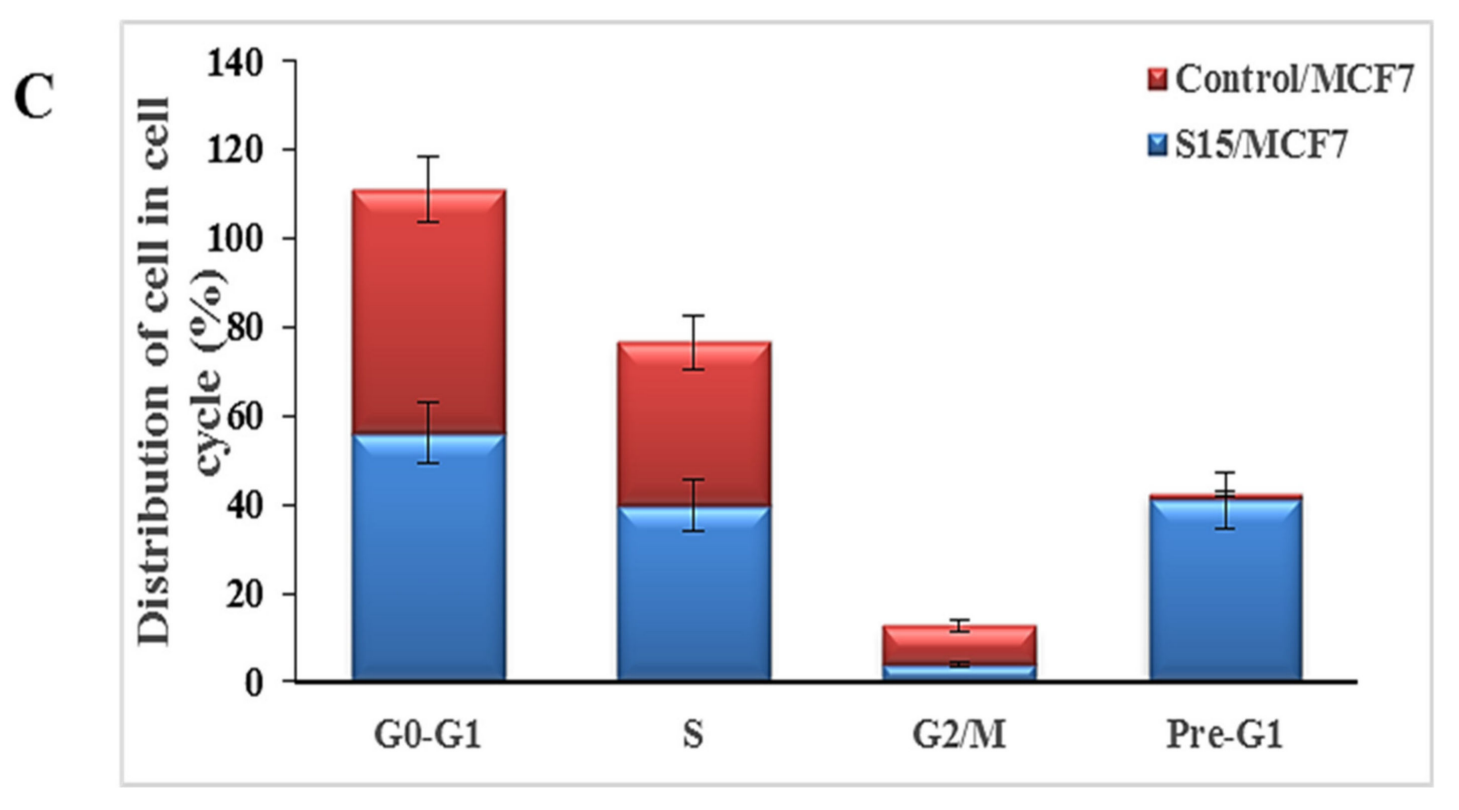
Percentage apoptosis and necrosis of MCF without treatment (**A**) or with **S15** treatment (**B**) on Effect of Compound **S15** on cell cycle distribution. Results were collected analyzed and blotted (**C**). The cells were treated with IC_50_ (μM) of compound **S15** and the cells were harvested and exposed to the analysis of cell-cycle. Data expressed as mean ± SEM.

The most potent compound, **S15**, was selected for cell cycle analysis and the initiation of apoptosis in the MCF7 cell line. The MCF7 cell line was incubated with an IC_50_ concentration for compound **S15** and its effects on the cell cycle profile and induction of apoptosis were inspected. Incubation of MCF7 cells with compounds **S15** resulted in an interference with the normal cell cycle distribution on this cell line. In MCF7, compound **S15** increased the percentage of cells in pre-G1 by more than 40-fold. The current results showed that compound **S15** resulted in arrest cell growth at G1/S (Table 4 and Figure 5). The initial G1/S checkpoint suggests that the cell is organized in order to start DNA replication and the damaged DNA is repaired. The increase in the number of the cells in the pre-G1 phase demonstrated a potential role of apoptosis (Table 4 and Figure 5).

**Figure 6:**
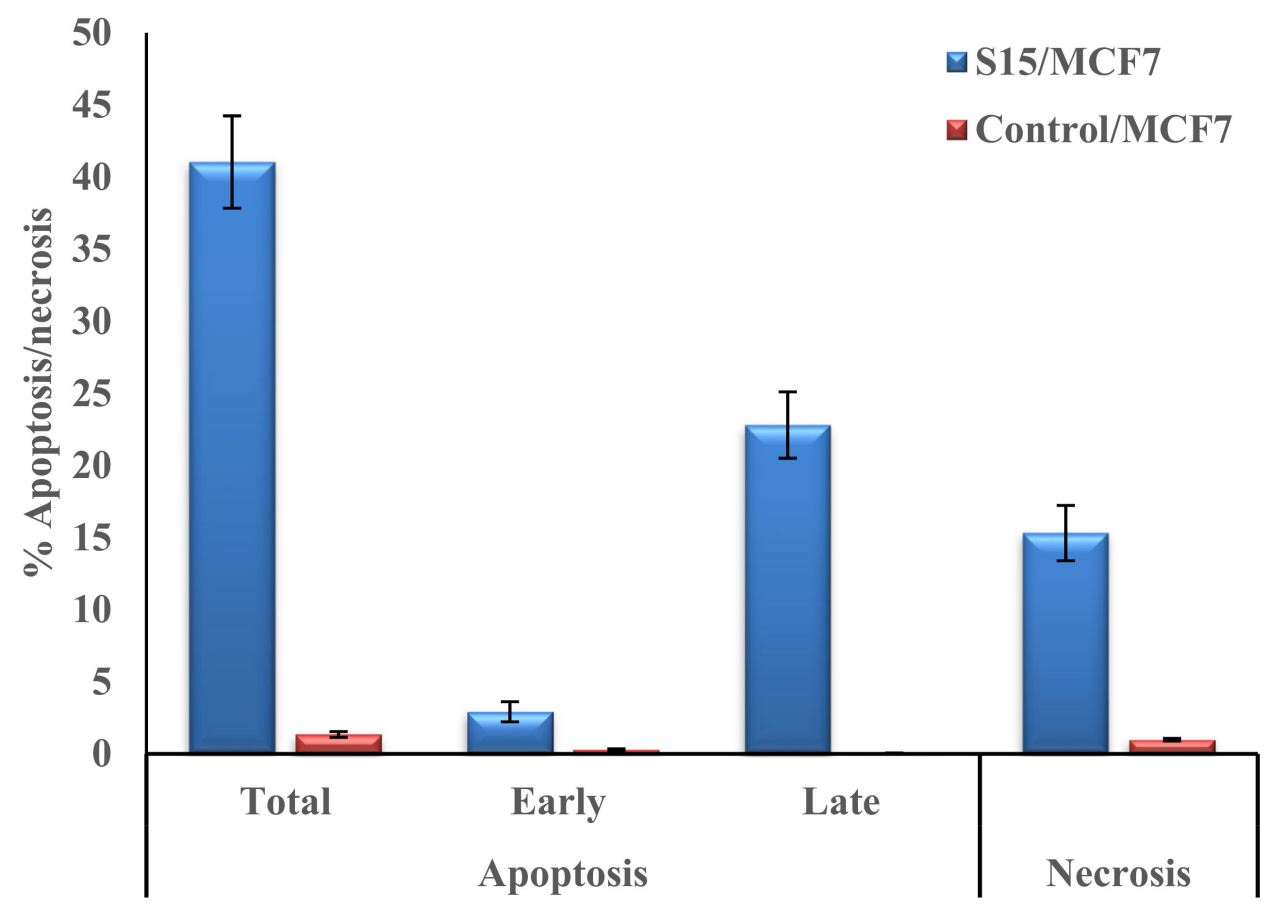
Effect of treatment of MCF7 cancer cell line with compound **S15** on the percentage cell necrosis and apoptosis. Data expressed as mean ± SEM.

Treatment of the MCF7 cancer cell line with compound **S15** increased the percentage of necrosis by 15-fold compared to control untreated MCF7. Interestingly, compound **S15** increased apoptosis by about 30-fold (Table 4 and Figure 6).

Inhibition of CA IX has been newly confirmed as a recent method for targeting hypoxic tumors [25]. CA IX play a critical role in regulating the pH of the breast cancer microenvironment, while CAXII is accompanied by a better prognosis, although both have the same catalytic function [26]. Furthermore, CA XII may endorse the invasion and migration of breast cancer cells [27]. In the current experiment, CA IX and CAXII inhibiting effects were implemented on 22 compounds using Dorzolamide HCL (DZM) as a reference standard. Out of 22 compounds, compounds **S2**, **S3**, **S8**, **S9** and **S15** showed superior inhibitory activity on CA IX and CA XII. Subsequently, effective compounds were then applied for CAI and CAII inhibitory activity. Earlier work has revealed that CA I has an important role in the migration of breast cancer [28]. Likewise, amplification of the CA1 gene was identified in nearly 25% studies on breast cancer [29]. On the other hand, in breast cancer CA II increased expression has been found to be allied with tumor progression, poor prognosis, and metastasis [30]. The current results showed that compounds **S2**, **S3**, **S8**, **S9** and **S15** have potent inhibitory effect on CA I and CAII, using acetazolamide as a reference standard. The current results confirm the superior inhibitory activity of compounds **S2**, **S3**, **S8**, **S9** and **S15** on CA I, CA II, CA IX and CA XII.

Breast cancer is the most commonly diagnosed cancer type in women. Hypoxic distributed regions are frequently identified in invasive breast cancer and is considered the worse type of cancer as it is metastatic and largely invasive [31]. Using hypoxia-effective anticancer is one of the prominent tactics to avoid hypoxic challenges [32]. The use of this strategy offers a discriminating tumor inhibition, providing lower toxicity to normoxic tissues. Although many trials have aimed to provide hypoxia-effective drugs for different tumor types, their activities against breast cancer are still low [33]. In order to confirm the possible anticancer activity, we selected compounds **S8**, **S9** and **S15** based on CAs assay. We explored the action of the derivatives on cell viability using breast cancer cell line, MCF7, below hypoxic and normoxic conditions and non-tumorigenic epithelial breast cell line, MCF10a, using Staurosporine as a reference standard. MTT assay displayed that compounds **S15** and **S8** reduced MCF7 cell viability more significantly under hypoxia than normoxia, with little effect on the cell viability of MCF10a, suggesting that our compounds could be promising hypoxia effective agents for breast cancer. We found in the current study that compounds **S15** triggered cell cycle arrest and apoptosis in G1/S of MCF7 cancer cells.

### 2.3. Molecular Docking Study

Carbonic anhydrases (Cas) are a group of Zn-metalloenzymes, in which Zn ions play a crucial role in its function [34,35]. Thus, drugs targeting such enzymes should engage in a coordination interaction with the Zn ion. Consequently, computational docking studies are utilized to accurately predict these kinds of interactions between ligands and Zn containing proteins.

In this regard, the most recently released version of *AutoDock Vina 1.2.0* is used to model and describe the Zn-coordinating ligands utilizing a specialized *AutoDock4Zn* forcefield [36,37,38]. This method does not describe only the energetic components of the interaction but also the geometric ones. This novel approach acts through creating a pseudo-atom (*TZ*) to describe the preferred position and the optimal tetrahedral coordination geometry of Zn ion complexed in proteins. Moreover, it defines the possibility for the interaction of Zn with coordinating elements such as nitrogen, oxygen and sulfur incorporated in the ligand. Additionally, the coordination geometry is encoded in the grid maps for the standard AutoDock4 (AD4) atom types. The new method includes an expanded forcefield, an exceedingly advanced scoring function, and can replicate the improved docking presentation related to an AD4 engine.

**Figure 7:**
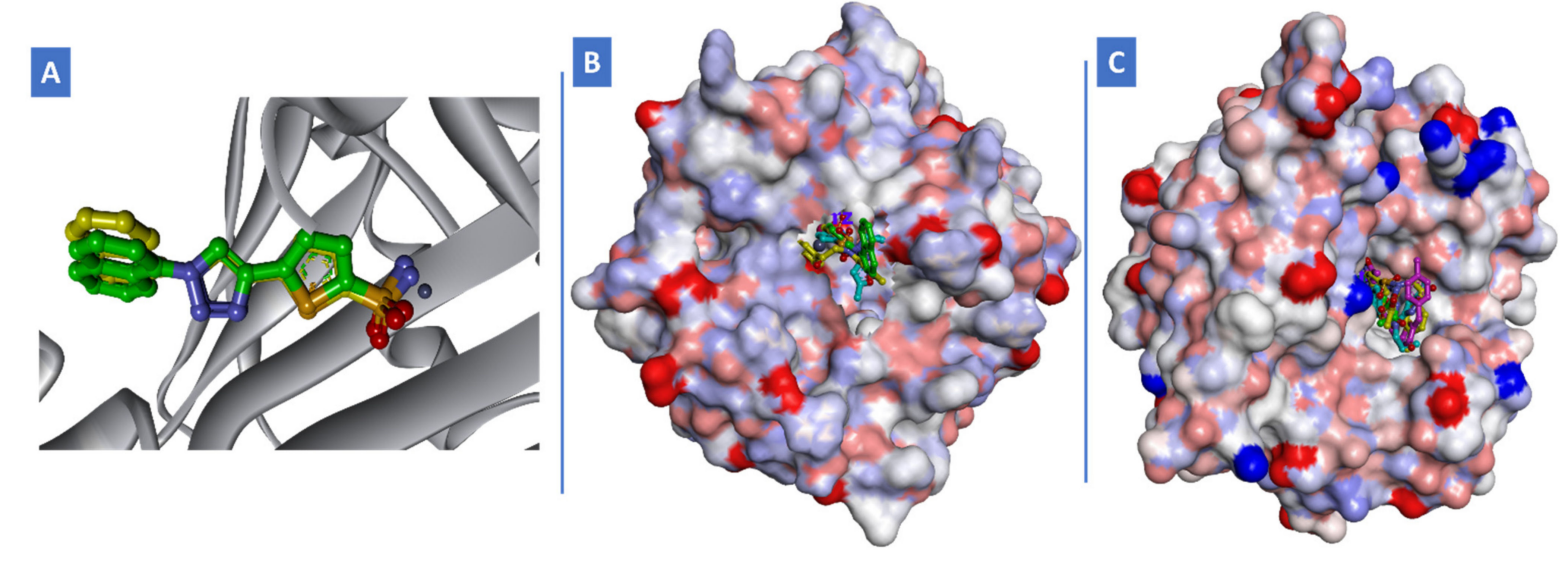
(**A**) ligand (9FK, green) co-crystallized and its redocked pose (yellow) inside the active place of *h*CA IX isoenzyme (PDB code: 5FL4); (**B**) Alignment of the top docked poses of compounds **S3** (cyan), **S15** (yellow) and **9FK** (green) as a co-crystalized ligand into the binding pocket of *h*CA IX isoenzyme (PDB code: 5FL4); (**C**) Alignment of the top docked poses of compounds **S3** (cyan), **S15** (yellow), **S19** (violet) and **acetazolamide** (green) as a co-crystalized ligand into the binding site of hCA-XII isoenzyme (PDB code: 1JD0). The protein structures were displayed as solvent surfaces colored by atom charge.

Utilizing this highly improved capability in *AutoDock Vina 1.2.0*, a docking study was conducted to delineate a plausible explanation for the binding patterns and key interactions adopted by the most active (**S3** and **S15**) inside the active site of tumor-associated isoenzymes CA-IX and compounds (**S15** and **S19**) inside the active site CA-XII. In doing so, the available 3D crystal structures of *h*CA-IX enzyme (Protein Data Bank (PDB): 5FL4, resolution = 1.82 Ǻ, complexed with 2-thiophene-sulfonamide ligand; **9FK**) and *h*CA-XII (PDB code: 1JD0, resolution = 1.50 Ǻ, complexed with acetazolamide) on the PDB (https://www.rcsb.org, accessed on 18 July 2022) were downloaded and used for this simulation. Initially, the docking procedure was validated by re-docking the co-crystallized ligand **9FK** into the active site of CA-IX and then inspected visually, and the RMSD was calculated. It revealed similar conformations and good alignment with low RMSD value of 0.63 Ǻ, Figure 1A. The superimposition of the highest docking poses of the greatest potent compounds; **S3** and **S15**, along with the co-crystallized ligands; **9FK** and acetazolamide into the active place of *h*CA-IX and *h*CA-XII, respectively, displays an amazing shape complementarity and similar orientations, Figure 7B,C.

**Figure 8:**
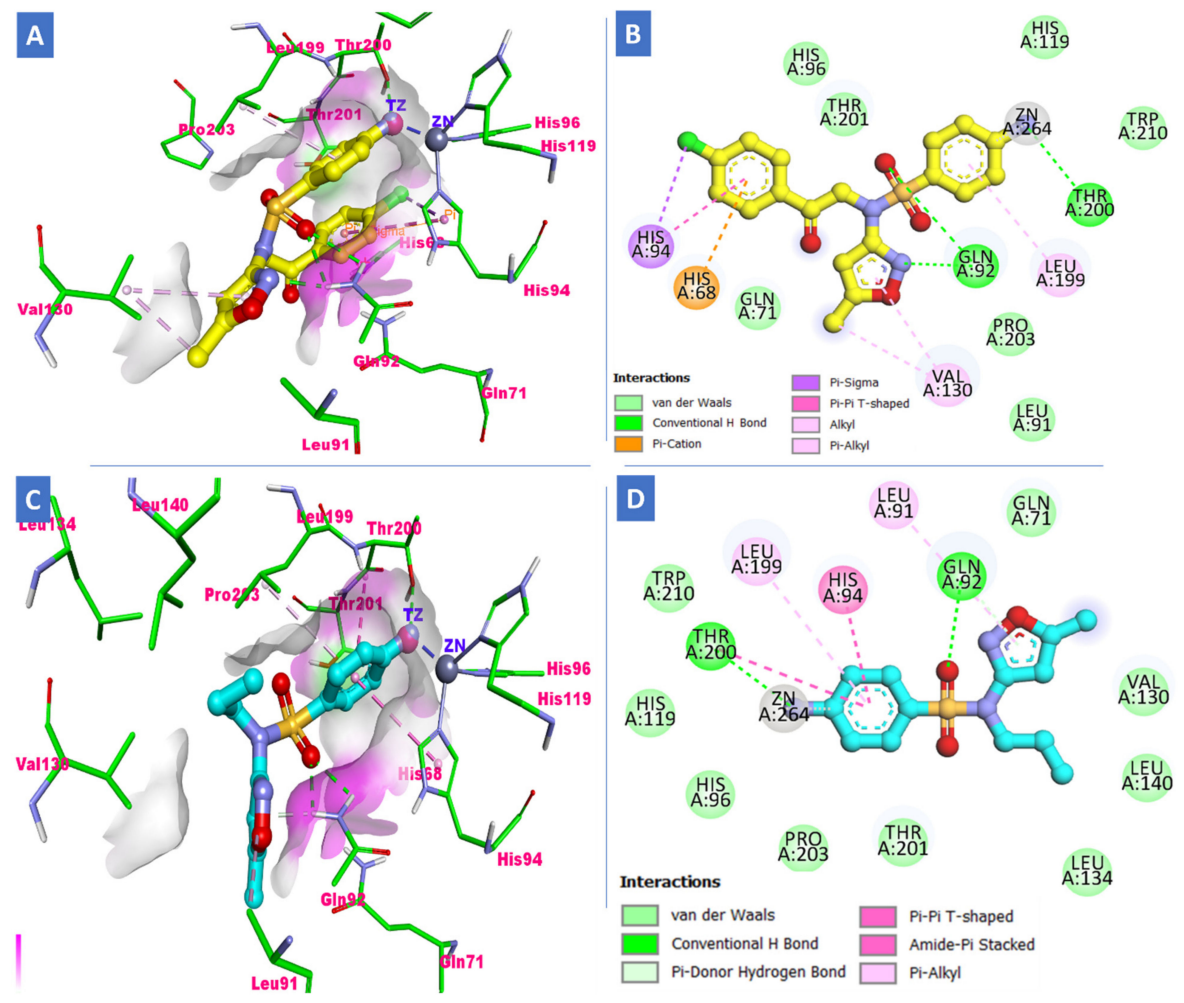
The docking results and interactions of compounds **S15** (yellow) and **S3** (cyan) into the active site of hCA-IX isoenzyme (PDB code: 5FL4); (**A**,**B**) 3D and 2D binding modalities of **S15**, respectively; (**C**,**D**) 3D and 2D binding modalities of **S3**, respectively. The docked poses were displayed in ball & stick style. Zn atom was shown as a blue ball while pseudoatom TZ was represented as a pink ball. Favorable co-ordinate bond with Zn cation, hydrogen bonds and π-Stacking were showed in dashed blue, green and orange dashed lines, respectively. Hydrogen bond surfaces around docked pos-es were created. Hydrogen atoms were omitted for clarity purpose. The alignment of the most dominant conformations of **S3**, **S15** and co-crystallized ligand, 9FK into the active place of CA-IX was shown in Figure 9A,B. All compounds maintained almost the same conformation in the active site. Interestingly, the visual inspection was in agreement with the obtained docking scores and were much higher than that of the redocked pose 9FK where compounds **S3** and **S15** recorded the *ΔG* values of −29.86, −28.98 kcal/mol, respectively compared with docking score of **9FK** with Δ*G* values of −17.87 kcal/mol.

Upon inspection of the docking results, it was revealed that the most potent compounds **S3** and **S15** (IC_50_ = 0.042 and 0.037 nM, respectively) bind within the CA-IX active site in a similar manner orientation compared to that of the co-crystalized ligand, **9FK,** where the anilino moiety was directed to the bottom of the active site near Zn cation, while their tails were allocated at the entrance of the catalytic binding pocket cleft engaging in some vital hydrophobic interactions with Leu91, Val130 and Leu140 residues. However, the coordinating points with Zn ion were different. In compounds **S3** and **S15,** the pseudoatom (TZ) was created on the N atom of amino group of the anilino moiety rather than NH of the sulfonamide moiety as in **9FK**. This shift could be explained by the increased length of the compounds’ tail and size compared to **9FK**. This amino group was also involved in an important H-bond interaction with Thr200 amino acid residue. Furthermore, the isoxazole ring in **S15** was involved in a set of hydrogen bonds with the well-known key residue Gln92, where sulfonamide group formed two H-bonds, while NH was involved in an additional H-bond. Moreover, the *p*-chlorophenyl ring was engaged in π-π stacking with the imidazole ring of His94 and π -cation interaction with His68 residue. Nevertheless, **S3** engaged in the same interactions, except one of the H-bond interactions of isoxazole with Gln92 residue, and this might be the reason behind the very slight decrease in activity. The 2D and 3D binding modes and key interactions of **S3** and **S15** against CA-IX isoform were depicted in Figure 8A–D and interaction results are illustrated in Table 5.

On the other hand, compound **S15** with an IC_50_ value of 0.06 nM and **S19** with lower activity (IC_50_ = 0.9 nM) were docked into the catalytic binding site of *h*CA-XII isoenzymes. The results showed that **S15** coordinates with the Zn cation with N atom of anilino moiety in the same manner that has been observed with *h*CA-IX. In addition, it was involved in a crucial H-bonding interaction with Thr199 residue. However, the two oxygens of the sulfonamide group were involved into two vital hydrogen bonds with Gln92 and Thr200 amino acids residues. Some hydrophobic interactions were noticed with Ala121, Val131, Leu141 and Leu198 residues, which greatly contributed to its stability inside the active pocket, Figure 10A,B. However, compound **S19** adopted a different orientation, where the pseudoatom (*TZ*) preferred to be on the NH of the sulfonamide, which resulted in some steric clashes, unfavorable coordination with Zn ion and unfavorable acceptor-acceptor interaction with Thr199 amino acid. This could explain the significant difference in activity compared to **S15**. Nevertheless, **S19** was engaged in other important H-bonding, hydrophobic, Van der Waal and pi-sulfur interactions, which significantly have a great impact on keeping its activity within the nanomolar range, Figure 10C,D. Compounds **S15** and **S19** recorded the Δ*G* values of −27.83 and −25.21 kcal/mol, respectively. Taken together, our in silico docking analysis strongly confirmed the potential inhibitory activity of our new hits against both hCA-IX and hCA-XII isoenzymes, which in turn might participate in the design and development of promising leads that could be utilized in the future as anticancer agents.

## 3. Materials and Methods

### 3.1. Chemistry

The instruments specifically used for NMR, IR, mass spectroscopy and elemental analysis of synthesis compounds are presented in the Supplementary Data (Section S2). Additionally, TLC plates and chemical supplies are illustrated in Section S2.

Derivatives **S5**, **S13a–d**, and **S18** were synthesized following known methods.

#### 3.1.1. General Method for Preparation of Sulfamethoxazole **S1–S4**

To a mixture of sulfamethoxazole (2.53 g, 10 mmol) and anhydrous potassium carbonate (5.52 g, 40 mmol) in dry DMF (30 mL), the corresponding alkyl chloride (10 mmol) was added dropwise. The reaction mixture was heated under reflux for 24 h then poured onto ice-cold water. The formed product was filtered, washed with water, dried and crystallized from absolute ethanol to give compounds **S1–S4**.

4-Amino-*N*-ethyl-*N*-(5-methylisoxazol-3-yl)benzenesulfonamide (**S1**):

Yield (84%); yellow crystals; m.p. 90–92 °C; IR (KBr): 3261 (NH) cm^−1^. ^1^HNMR at *δ* 1.16 (t, *J* = 6.8 Hz, 3H, CH_2_-C**H_3_**); 2.35 (s, 3H, CH_3_ isoxazole ring); 3.70 (q, *J* = 6.8 Hz, 2H, CH_2_); 6.19 (s, 2H, NH_2_); 6.41 (s, 1H, isoxazole H-4); 6.61 (d, *J* = 8.8 Hz, 2H, benzenesulfonamide H-3,5); 7.44 (d, *J* = 8.8 Hz, 2H, benzenesulfonamide H-2,6). ^13^CNMR at *δ*: 12.57 (CH_3_ isoxazole), 14.18 (CH_3_), 43.74 (CH_2_), 97.86 (isoxazole C-4), 113.29, 122.85, 129.44, 154.24, 159.72, 170.71. EIMS (*m/z*) 281.41 M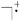 (11.27%), 268.41 (100%). Anal. Calcd. for C_12_H_15_N_3_O_3_S: C, 51.23; H, 5.37; N, 14.94; Found; C, 51.61; H, 5.02; N, 14.86.

*N*-Allyl-4-amino-*N*-(5-methylisoxazol-3-yl)benzenesulfonamide (**S2**):

Yield (83%); yellowish white powder; m.p. 105–107 °C; IR (KBr): 3345 (NH) cm^−1^. ^1^HNMR at *δ*: 2.34 (s, 3H, CH_3_ isoxazole ring); 4.30 (d, *J* = 5.2 Hz, 2H, NH-C**H**_2_); 5.20 (dd, *J* = 1.2, 17.2 Hz, 2H, CH=C**H**_2_); 5.75–5.85 (m, 1H, CH allyl); 6.22 (s, 2H, NH_2_); 6.40 (s, 1H, isoxazole H-4); 6.62 (d, *J* = 8.4 Hz, 2H, benzenesulfonamide H-3, 5); 7.48 (d, *J* = 8.4 Hz, 2H, benzenesulfonamide H-2,6). ^13^CNMR at *δ*: 12.63 (CH_3_ isoxazole), 50.57 (NH-CH_2_), 97.82 (isoxazole C-4), 113.25, 116.38, 118.14, 122.55, 129.60, 133.14, 154.25, 159.79, 170.85. EIMS (*m/z*) 293.84 M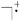 (15.73%), 166.27 (100%). Anal. Calcd. for C_13_H_15_N_3_O_3_S: C, 53.23; H, 5.15; N, 14.32; Found; C, 53.11; H, 5.22; N, 14.06.

4-Amino-*N*-(5-methylisoxazol-3-yl)-*N*-propylbenzenesulfonamide (**S3**)

Yield (85%); white crystals; m.p. 75–77 °C; IR (KBr): 3330 (NH) cm^−1^. ^1^HNMR at *δ*: 0.85 (t, *J* = 8.0 Hz, 3H, CH_2_-C**H_3_**); 1.54–1.63 (m, 2H, C**H_2_**-CH_3_); 2.35 (s, 3H, CH_3_ isoxazole ring); 3.59 (t, *J* = 8.0 Hz, 2H, NH-CH_2_); 6.20 (s, 2H, NH_2_); 6.41 (s, 1H, isoxazole H-4); 6.60 (d, *J* = 8.0 Hz, 2H, benzenesulfonamide H-3, 5); 7.42 (d, *J* = 8.0 Hz, 2H, benzenesulfonamide H-2, 6). ^13^CNMR at *δ*: 11.40 (CH_3_), 12.60 (CH_3_ isoxazole), 21.48 (**C**H_2_CH_3_), 50.02 (NH-CH_2_), 98.09 (isoxazole C-4), 113.26, 122.66, 129.45, 154.22, 159.95, 170.74. EIMS (*m/z*) 295.00 M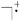 (21.00%), 63.17 (100%). Anal. Calcd. for C_13_H_17_N_3_O_3_S: C, 52.87; H, 5.80; N, 14.23; Found; C, 52.91; H, 6.02; N, 14.15.

4-Amino-*N*-butyl-*N*-(5-methylisoxazol-3-yl)benzenesulfonamide (**S4**)

Yield (79%); white crystals; m.p. 85–87 °C; IR (KBr): 3230 (NH) cm^−1^. ^1^HNMR at *δ*: 0.86 (t, *J* = 7.6 Hz, 3H, CH_2_-C**H_3_**); 1.25–1.30 (m, 2H, C**H_2_**-CH_3_); 1.52–1.56 (m, 2H, -CH_2_-C**H_2_**-CH_2_); 2.35 (s, 3H, CH_3_ isoxazole ring); 3.62 (t, *J* = 7.6 Hz, 2H, NH-CH_2_); 6.19 (s, 2H, NH_2_); 6.40 (s, 1H, isoxazole H-4); 6.60 (d, *J* = 8.4 Hz, 2H, benzenesulfonamide H-3, 5); 7.41 (d, *J* = 8.4 Hz, 2H, benzenesulfonamide H-2,6). ^13^CNMR at *δ*: 12.58 (CH_3_ isoxazole), 13.95 (CH_3_), 19.73 (**C**H_2_CH_3_), 30.23(CH_2_-**C**H_2_-CH_2_), 48.17, (NH-CH_2_), 98.15 (isoxazole C-4), 113.28, 122.64, 129.44, 154.19, 159.94, 170.78. EIMS (*m/z*) 309.79 M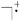 (12.93%), 189.82 (100%). Anal. Calcd. for C_14_H_19_N_3_O_3_S: C, 54.35; H, 6.19; N, 13.58; Found; C, 54.23; H, 6.07; N, 13.66.

#### 3.1.2. General Method for Preparation of Compounds **S6–S11**

A solution of sulfamethoxazole (2.53 g, 10 mmol) in dry DMF (30 mL) was cooled to 0–5 °C. The appropriate acid chloride (10 mmol) was added slowly with vigorous stirring, followed by the addition of triethylamine (0.5 mL). The reaction mixture was then stirred at *rt* for 24 h (for compounds **S6** and **S7**) or heated under reflux (for compounds **S8–S11**). At the end of the reaction, the mixture was poured into ice-cooled water and the precipitate was filtered off, dried and crystallized from methanol to afford the target compounds **S6–S11**.

3-Chloro-*N*-(4-(*N*-(5-methylisoxazol-3-yl)sulfamoyl)phenyl)propanamide (**S6**)

Yield (82%); pale yellow powder; m.p. 208–210 °C; IR (KBr): 3352 (NH), 1675 (C=O) cm^−1^. ^1^HNMR at *δ*: 2.29 (s, 3H, CH_3_ isoxazole ring); 2.87 (t, *J* = 6.4 Hz, 2H, CO-CH_2_); 3.88 (t, *J* = 6.4 Hz, 2H, CH_2_-Cl); 6.13 (s, 1H, isoxazole H-4); 7.78–7.83 (m, 4H, phenyl H-2, 3, 5, 6); 10.51 (s, 1H, NH); 11.34 (s, 1H, NH). ^13^CNMR at *δ*: 12.52 (CH_3_ isoxazole), 39.33 (**C**H_2_CO), 41.00 (CH_2_-Cl), 95.83 (isoxazole C-4), 119.35, 129.30, 133.68, 143.61, 158.01, 169.30 (CO), 170.78. EIMS (*m/z*) 343.82 M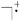 (26.11%), 309.58 (100%). Anal. Calcd. for C_13_H_14_ClN_3_O_4_S: C, 45.42; H, 4.10; N, 12.22; Found; C, 45.71; H, 4.18; N, 12.37.

4-Chloro-*N*-(4-(*N*-(5-methylisoxazol-3-yl)sulfamoyl)phenyl)butanamide (**S7**)

Yield (77%); white powder; m.p. 212–214 °C; IR (KBr): 3303 (NH), 1682 (C=O) cm^−1^. ^1^HNMR at *δ*: 2.00–2.07 (m, 2H, CH_2_-C**H_2_**-CH_2_); 2.29 (s, 3H, CH_3_ isoxazole ring); 2.53 (t, *J* = 7.6 Hz, 2H, CO-CH_2_); 3.70 (t, *J* = 6.4 Hz, 2H, CH_2_-Cl); 6.13 (s, 1H, isoxazole H-4); 7.79–7.82 (m, 4H, phenyl H-2, 3, 5, 6); 10.40 (s, 1H, NH); 11.32 (s, 1H, NH). ^13^CNMR at *δ*: 12.51 (CH_3_ isoxazole), 28.12 (CH_2_-**C**H_2_-CH_2_), 33.94 (**C**H_2_CO), 45.38 (CH_2_-Cl), 95.83 (isoxazole C-4), 119.27, 129.29, 133.38, 143.90, 158.03, 170.75 (CO), 171.49. EIMS (*m/z*) 357.07 M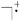 (26.08%), 87.68 (100%). Anal. Calcd. for C_14_H_16_ClN_3_O_4_S: C, 47.00; H, 4.51; N, 11.74; Found; C, 47.23; H, 4.22; N, 11.67.

4-Methyl-*N*-(4-(*N*-(5-methylisoxazol-3-yl)sulfamoyl)phenyl)benzamide (**S8**)

Yield (73%); yellowish white powder; m.p. 225–227 °C; IR (KBr): 3283 (NH), 1666 (C=O) cm^−1^. ^1^HNMR at *δ*: 2.28 (s, 3H, CH_3_ isoxazole ring); 2.39 (s, 3H, CH_3_); 6.10 (s, 1H, isoxazole H-4); 7.35 (d, *J* = 7.6 Hz, 2H, benzamide H-3,5); 7.80–7.96 (m, 6H, phenyl H-2, 3, 5,6 and benzamide H-2, 6); 10.51 (s, 1H, NH); 11.72 (s, 1H, NH). ^13^CNMR at *δ*: 12.57 (CH_3_ isoxazole), 21.50 (CH_3_), 96.13 (isoxazole C-4), 120.38, 128.16, 128.31, 129.48, 131.95, 142.63, 143.59, 166.37. EIMS (*m/z*) 371.62 M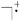 (11.27%), 285.69 (100%). Anal. Calcd. for C_18_H_17_N_3_O_4_S: C, 58.21; H, 4.61; N, 11.31; Found; C, 58.40; H, 4.88; N, 11.16. 

4-Chloro-*N*-(4-(*N*-(5-methylisoxazol-3-yl)sulfamoyl)phenyl)benzamide (**S9**)

Yield (79%); yellow crystals; m.p. 233–235 °C; IR (KBr): 3242 (NH), 1673 (C=O) cm^−1^. ^1^HNMR at *δ*: 2.30 (s, 3H, CH_3_ isoxazole ring); 6.16 (s, 1H, isoxazole H-4); 7.63 (d, *J* = 8.4 Hz, 2H, benzamide H-3, 5); 7.87 (d, *J* = 8.8 Hz, 2H, phenyl H-3, 5); 7.98–8.00 (m, 4H, phenyl H-2, 6 and benzamide H-2, 6); 10.69 (s, 1H, NH); 11.38 (s, 1H, NH). ^13^CNMR at *δ*: 12.53 (CH_3_ isoxazole), 95.88 (isoxazole C-4), 113.06, 120.57, 128.38, 128.68, 129.04, 130.27, 133.51, 133.54, 134.15, 137.36, 143.82, 158.05, 165.50 (CO), 170.77. EIMS (*m/z*) 391.73 M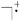 (61.57%), 60.02 (100%). Anal. Calcd. for C_17_H_14_N_4_O_6_S: C, 50.74; H, 3.51; N, 13.92; Found; C, 51.01; H, 3.32; N, 13.64.

*N*-(4-(*N*-(5-Methylisoxazol-3-yl)sulfamoyl)phenyl)-4-nitrobenzamide (**S10**)

Yield (73%); yellow crystals; m.p. 188–190 °C; IR (KBr): 3325 (NH), 1681 (C=O) cm^−1^. ^1^HNMR at *δ*: 2.30 (s, 3H, CH_3_ isoxazole ring); 6.16 (s, 1H, isoxazole H-4); 7.89 (d, *J* = 8.8 Hz, 2H, phenyl H-3, 5); 8.00 (d, *J* = 8.8 Hz, 2H, phenyl H-2, 6); 8.19 (d, *J* = 8.4 Hz, 2H, nitrobenzamide H-2,6); 8.39 (d, *J* = 8.4 Hz, 2H, nitrobenzamide H-3, 5); 10.94 (s, 1H, NH); 11.41 (s, 1H, NH). ^13^CNMR at *δ*: 12.52 (CH_3_ isoxazole), 95.88 (isoxazole C-4), 113.06, 120.72, 124.08, 128.44, 129.30, 129.86, 134.51, 140.48, 143.51, 149.84, 158.03, 165.00 (CO), 170.80. EIMS (*m/z*) 402.81 M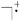 (66.30%), 243.77 (100%). Anal. Calcd. for C_17_H_14_ClN_3_O_4_S: C, 52.11; H, 3.60; N, 10.72; Found; C, 52.03; H, 3.92; N, 10.56.

3,4,5-Trimethoxy-*N*-(4-(*N*-(5-methylisoxazol-3-yl)sulfamoyl)phenyl)benzamide (**S11**)

Yield (69%); white powder; m.p. 242–244 °C; IR (KBr): 3295 (NH), 1687 (C=O) cm^−1^. ^1^HNMR at *δ*: 2.31 (s, 3H, CH_3_ isoxazole ring); 3.74 (s, 3H, OCH_3_); 3.87 (s, 6H, 2 OCH_3_); 6.15 (s, 1H, isoxazole H-4); 7.27 (s, 2H, benzamide H-2, 6); 7.86 (d, *J* = 8.4 Hz, 2H, phenyl H-3,5); 7.96 (d, *J* = 8.4 Hz, 2H, phenyl H-2,6); 10.49 (s, 1H, NH); 11.37 (s, 1H, NH). ^13^CNMR at *δ*: 12.52 (CH_3_ isoxazole), 56.59 (2 OCH_3_), 60.64 (OCH_3_), 95.88 (isoxazole C-4), 105.93, 120.67, 128.35, 129.92, 134.05, 141.11, 143.87, 153.13, 158.12, 165.99 (CO), 170.77. EIMS (*m/z*) 447.01 M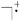 (39.39%), 402.65 (100%). Anal. Calcd. for C_20_H_21_N_3_O_7_S: C, 53.69; H, 4.73; N, 9.39; Found; C, 53.41; H, 4.85; N, 9.57.

#### 3.1.3. Preparation Method of Compounds **S12** and **S14–S17**

A mixture of sulphamethoxazole (2.53 g, 10 mmol), 3-chloropropiophenone or compounds **S13a–d** (10 mmol) and triethylamine (0.5 mL) in dry dimethylformamide (30 mL) was heated under reflux for 24 h. The reaction mixture was poured into ice-cooled water, the precipitate was filtered, dried and crystallized from methanol to obtain the target compounds **S12** and **S14–S17**.

4-Amino-N-(5-methylisoxazol-3-yl)-*N*-(3-oxo-3-phenylpropyl)benzenesulfonamide (**S12**)

Yield (64%); pale yellow powder; m.p. 200–202 °C; IR (KBr): 3331 (NH_2_), 1765 (C=O) cm^−1^. ^1^HNMR, at *δ*: 2.29 (s, 3H, CH_3_ isoxazole ring); 3.33 (t, *J* = 6.0 Hz, 2H, CO-CH_2_); 3.46 (t, *J* = 6.0 Hz, 2H, CH_2_-N); 6.11 (s, 2H, NH_2_); 6.64–6.70 (m, 3H, benzenesulfonamide H-3,5 and oxazole CH); 7.51–7.56 (m, 4H, benzenesulfonamide H-2,6 phenyl H-3, 5); 7.64 (t, *J* = 7.6, 1H, phenyl H-4); 7.98 (d, *J* = 7.2, 2H, phenyl H-2,6); ^13^CNMR at *δ*: 12.51 (CH_3_ isoxazole), 37.72 (**C**H_2_CO), 39.71 (CH_2_-N), 95.75 (isoxazole C-4), 111.36, 124.68, 128.38, 129.20, 129.24, 133.75, 137.01, 152.84, 158.44, 170.38, 198.86 (CO). EIMS (*m/z*) 385.54 M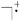 (33.65%), 204.82 (100%). Anal. Calcd. for C_19_H_19_N_3_O_4_S: C, 59.21; H, 4.97; N, 10.90; Found; C, 59.33; H, 4.87; N, 10.76.

4-Amino-*N*-(5-methylisoxazol-3-yl)-*N*-(2-oxo-2-(p-tolyl)ethyl)benzenesulfonamide (**S14**)

Yield (66%); yellow powder; m.p. 180–182 °C; IR (KBr): 3355, 3262 (NH_2_), 1673 (C=O) cm^−1^. ^1^HNMR at *δ*: 2.25 (s, 3H, CH_3_); 2.34 (s, 3H, CH_3_ isoxazole ring); 4.49 (s, 2H, CH_2_); 6.25 (s, 2H, NH_2_); 6.41 (s, 1H, isoxazole H-4); 6.63 (d, *J* = 8.8 Hz, 2H, tolyl H-3,5); 7.11 (d, *J* = 8.4 Hz, 2H, phenyl H-2,6); 7.45 (d, *J* = 8.4 Hz, 2H, phenyl H-3,5); 7.55 (d, *J* = 8.8 Hz, 2H, tolyl H-2,6); 10.06 (s, 1H, NH). ^13^CNMR, at *δ*: 12.58 (CH_3_ isoxazole), 21.15 (CH_3_), 50.66 (CH_2_), 97.18 (isoxazole C-4), 113.23, 119.48, 122.43, 129.64, 129.89, 132.73, 136.78, 154.45, 160.02, 165.27 (CO), 170.82. EIMS (*m/z*) 400.26 M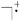 (26.30%), 211.22 (100%). Anal. Calcd. for C_19_H_20_N_4_O_4_S: C, 56.99; H, 5.03; N, 13.99; Found; C, 56.41; H, 4.89; N, 14.16.

4-Amino-N-(2-(4-chlorophenyl)-2-oxoethyl)-N-(5-methylisoxazol-3-yl)benzenesulfonamide (**S15**)

Yield (75%); brown powder; m.p. 182–184 °C; IR (KBr): 3333, 3281 (NH), 1661 (C=O) cm^−1^. ^1^HNMR at *δ*: 2.34 (s, 3H, CH_3_ isoxazole ring); 4.50 (s, 2H, CH_2_); 6.25 (s, 2H, NH_2_); 6.41 (s, 1H, isoxazole H-4); 6.62 (d, *J* = 8.8 Hz, 2H, phenyl H-2,6); 7.37 (d, *J* = 8.8 Hz, 2H, chlorophenyl H-3,5); 7.53 (d, *J* = 8.8 Hz, 2H, phenyl H-3,5); 7.58 (d, *J* = 8.8 Hz, 2H, chlorophenyl H-2,6); 10.29 (s, 1H, NH). ^13^CNMR at *δ*: 12.58 (CH_3_ isoxazole), 50.69 (CH_2_), 97.08 (isoxazole C-4), 113.23, 121.05, 122.31, 127.41, 129.20, 129.89, 138.16, 154.45, 159.95, 165.74 (CO), 170.95. EIMS (*m/z*) 420.99 M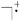 (97.40%), 402.69 (100%). Anal. Calcd. for C_18_H_17_ClN_4_O_4_S: C, 51.37; H, 4.07; N, 13.31; Found; C, 51.50; H, 4.19; N, 13.38.

4-Amino-N-(5-methylisoxazol-3-yl)-N-(2-(4-nitrophenyl)-2-oxoethyl)benzenesulfonamide (**S16**)

Yield (72%); yellowish brown powder; m.p. 186–188 °C; IR (KBr): 3354, 3221 (NH_2_), 1654 (C=O) cm^−1^. ^1^HNMR at *δ*: 2.34 (s, 3H, CH_3_ isoxazole ring); 4.59 (s, 2H, CH_2_); 6.27 (s, 2H, NH_2_); 6.43 (s, 1H, isoxazole H-4); 6.64 (d, *J* = 8.4 Hz, 2H, phenyl H-2,6); 7.557 (d, *J* = 8.4 Hz, 2H, phenyl H-3,5); 7.81 (d, *J* = 8.8 Hz, 2H, nitrophenyl H-2,6); 8.23 (d, *J* = 8.8 Hz, 2H, nitrophenyl H-3,5); 10.83 (s, 1H, NH). ^13^CNMR at *δ*: 12.59 (CH_3_ isoxazole), 50.90 (CH_2_), 97.03 (isoxazole C-4), 113.25, 119.26, 122.20, 125.32, 130.54, 142.78, 145.36, 154.53, 159.92, 166.65 (CO), 171.05. EIMS (*m/z*) 431.54 M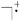 (25.98%), 231.96 (100%). Anal. Calcd. for C_18_H_17_N_5_O_6_S: C, 50.11; H, 3.97; N, 16.23; Found; C, 50.23; H, 4.09; N, 16.48.

4-Amino-*N*-(5-methylisoxazol-3-yl)-*N*-(2-oxo-2-(3,4,5-trimethoxyphenyl)ethyl) benzenesulfonamide (**S17**)

Yield (64%); pale yellow powder; m.p. 201–203 °C; IR (KBr): 3291, 3236 (NH_2_), 1667 (C=O) cm^−1^. ^1^HNMR at *δ*: 2.34 (s, 3H, CH_3_ isoxazole ring); 3.68 (s, 3H, OCH_3_); 3.74 (s, 6H, 2 OCH_3_); 4.47 (s, 2H, CH_2_); 6.24 (s, 2H, NH_2_); 6.41 (s, 1H, isoxazole H-4); 6.63 (d, *J* = 8.4 Hz, 2H, phenyl H-2,6); 6.95 (s, 2H, trimethoxyphenyl H-2,6); 7.52 (d, *J* = 8.4 Hz, 2H, phenyl H-3,5); 10.09 (s, 1H, NH). ^13^CNMR at *δ*: 12.59 (CH_3_ isoxazole), 50.67 (CH_2_), 56.16 (2 OCH_3_), 60.59 (OCH_3_), 97.18 (isoxazole C-4), 113.24, 122.44, 129.82, 133.91, 135.43, 153.23, 154.46, 160.00, 165.36 (CO), 170.87. EIMS (*m/z*) 476.18 M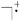 (15.15%), 384.64 (100%). Anal. Calcd. for C_21_H_24_N_4_O_7_S: C, 52.93; H, 5.08; N, 11.76; Found; C, 52.64; H, 4.99; N, 11.56.

#### 3.1.4. Method for Preparation of Benzenesulfonamides **S19–S22**

A mixture of thiazol-2-ylamino benzenesulfonamide derivative **S18** (3.52 g, 10 mmol), appropriate aromatic aldehyde (10 mmol) and CH_3_COONa (1.23 g, 15 mmol) in glacial acetic acid (20 mL) was heated under reflux for 24 h. The mixture was poured onto crushed ice, and the precipitate was filtered, dried, and crystallized from aqueous ethanol 95% to give the target products **S19–S22**.

(*Z*)-4-((5-(4-Methylbenzylidene)-4-oxo-4,5-dihydrothiazol-2-yl)amino)-*N*-(5-methylisoxazol-3-yl)benzenesulfonamide (**S19**)

Yield (86%); white powder; m.p. 228–230 °C; IR (KBr): 3362 (NH), 1753 (C=O) cm^−1^. ^1^HNMR at *δ*: 2.32 (s, 6H, 2 CH_3_); 6.18 (s, 1H, isoxazole H-4); 7.25–7.52 (m, 7H, benzenesulfonamide H-2,3,5,6, methylbenzylidene H-3,5 and NH); 7.65–7.89 (m, 3H, methylbenzylidene H-2,6 and CH); 11.97 (s, 1H, NH). ^13^CNMR at *δ*: 12.55 (CH_3_ isoxazole), 21.54 (CH_3_), 95.96 (isoxazole C-4), 122.47, 128.91, 130.28, 130.38, 130.73, 135.75, 140.74, 158.08, 170.83. EIMS (*m/z*) 456.94 M + 1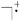 (7.45%), 250.90 (100%). Anal. Calcd. for C_21_H_18_N_4_O_4_S_2_: C, 55.49; H, 3.99; N, 12.33; Found; C, 55.36; H, 3.61; N, 12.66.

(*Z*)-4-((5-(4-Chlorobenzylidene)-4-oxo-4,5-dihydrothiazol-2-yl)amino)-*N*-(5-methylisoxazol-3-yl)benzenesulfonamide (**S20**)

Yield (75%); yellow crystals; m.p. 275–277 °C; IR (KBr): 3295 (NH), 1711 (C=O) cm^−1^. ^1^HNMR at *δ*: 2.32 (s, 3H, CH_3_ isoxazole); 6.18 (s, 1H, isoxazole H-4); 7.25 (d, *J* = 7.2 Hz, 2H, benzenesulfonamide H-3,5); 7.55–7.68 (m, 5H, benzenesulfonamide H-2,6, chlorobenzylidene H-3,5 and NH); 7.87–7.97 (m, 3H, chlorobenzylidene H-2,6 and CH); 11.56 (s, 1H, NH). ^13^CNMR at *δ*: 12.55 (CH_3_ isoxazole), 95.93 (isoxazole C-4), 122.47, 128.96, 129.84, 130.38, 131.86, 135.07, 140.74, 158.01, 170.89. EIMS (*m/z*) 474.21 M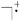 (20.45%), 344.74 (100%). Anal. Calcd. for C_20_H_15_ClN_4_O_4_S_2_: C, 50.58; H, 3.18; N, 11.80; Found; C, 50.66; H, 3.44; N, 12.06.

(*Z*)-*N*-(5-Methylisoxazol-3-yl)-4-((5-(4-nitrobenzylidene)-4-oxo-4,5-dihydrothiazol-2-yl)amino)benzenesulfonamide (**S21**)

Yield (69%); yellow powder; m.p. 266–268 °C; IR (KBr): 3235 (NH), 1706 (C=O) cm^−1^. ^1^HNMR at *δ*: 2.32 (s, 3H, CH_3_ isoxazole); 6.18 (s, 1H, isoxazole H-4); 7.26 (d, *J* = 8 Hz, 2H, benzenesulfonamide H-3,5); 7.77–8.05 (m, 5H, benzenesulfonamide H-2,6, nitrobenzylidene H-2,6 and NH); 8.27–8.36 (m, 3H, nitrobenzylidene H-3,5 and CH); 11.95 (s, 1H, NH). ^13^CNMR at *δ*: 12.54 (CH_3_ isoxazole), 95.93 (isoxazole C-4), 121.41, 122.46, 124.75, 128.00, 128.80, 129.01, 129.84, 131.14, 135.96, 139.97, 147.66, 158.00, 170.91. EIMS (*m/z*) 485.49 M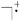 (24.56%), 253.28 (100%). Anal. Calcd. for C_20_H_15_N_5_O_6_S_2_: C, 49.48; H, 3.11; N, 14.43; Found; C, 49.83; H, 3.53; N, 14.76.

(*Z*)-*N*-(5-Methylisoxazol-3-yl)-4-((4-oxo-5-(3,4,5-trimethoxybenzylidene)-4,5-dihydrothiazol-2-yl)amino)benzenesulfonamide (**S22**)

Yield (71%); yellowish brown crystals; m.p. 240–242 °C; IR (KBr): 3382 (NH), 1723 (C=O) cm^−1^. ^1^HNMR at *δ*: 2.31 (s, 3H, CH_3_ isoxazole); 3.72 (s, 6H, 2 OCH_3_), 3.77 (s, 3H, OCH_3_), 6.18 (s, 1H, isoxazole H-4); 6.81–7.26 (m, 3H, benzenesulfonamide H-3,5 and NH); 7.64–7.97 (m, 5H, trimethoxybenzylidene H-2,6, benzenesulfonamide H-2,6 and CH); 11.47 (s, 1H, NH). ^13^CNMR at *δ*): 12.52 (CH_3_ isoxazole), 56.42 (2 OCH_3_), 60.67 (OCH_3_), 95.95 (isoxazole C-4), 107.21, 107.79, 120.94, 122.49, 122.47, 128.82, 130.98, 132.44, 135.40, 139.68, 153.64, 157.98, 170.86. EIMS (*m/z*) 530.65 M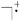 (29.89%), 49.79 (100%). Anal. Calcd. for C_23_H_22_N_4_O_7_S_2_: C, 52.07; H, 4.18; N, 10.56; Found; C, 51.86; H, 4.41; N, 10.79.

### 3.2. Biological Activities

#### 3.2.1. Materials and Methods

##### Chemicals and Kits

The specifications and suppliers of the chemicals and kits are presented in Supplementary Section S2 (attached file).

#### 3.2.2. CA IX Inhibitory Assay

The *h*CA IX was assayed following the manufacturer’s instruction and reported method. (Table 1, Figure 3A,B) [39] (Supplementary Material Section S2).

#### 3.2.3. hCA XII Inhibitory Assay

The *h*CA XII was assayed following the manufacturer’s instruction and reported method [40] (Supplementary Material Section S2) (Table 1, Figure 3A,B).

#### 3.2.4. CA1 Inhibitory Assay

The *h*CA I was assayed following the manufacturer’s instruction and reported method [41] (Supplementary Material Section S2) (Table 2).

#### 3.2.5. CAII Inhibitory Assay

CAII was assayed following the manufacturer’s instruction and reported method [42] (Supplementary Material Section S2) (Table 2).

#### 3.2.6. Cell Culture Protocol

Suppling, specification and incubation of MCF7 and MCF10a cell lines were carried out according to the method mentioned in detail in Supplementary Material Section S2.

#### 3.2.7. MTT–Cytotoxicity Assay Protocol

MTT assay is a method used for in vitro cytotoxicity of different compounds. The method details are illustrated in Supplementary Material Section S2. (Table 3, Figure 4).

#### 3.2.8. Annexin V-FITC Assay for Apoptosis

Treated MCF7 and MCF10a cells of 1–5 × 105 were collected and suspended with binding buffer and cultured and incubated with 5 μL of both propidium iodide (PI 50mg/mL) and Annexin V-FITC. The details regarding the method of detection are listed in Supplementary Material Section S2. (Table 4, Figure 5 and Figure 6).

### 3.3. Docking Study

**Figure 9:**
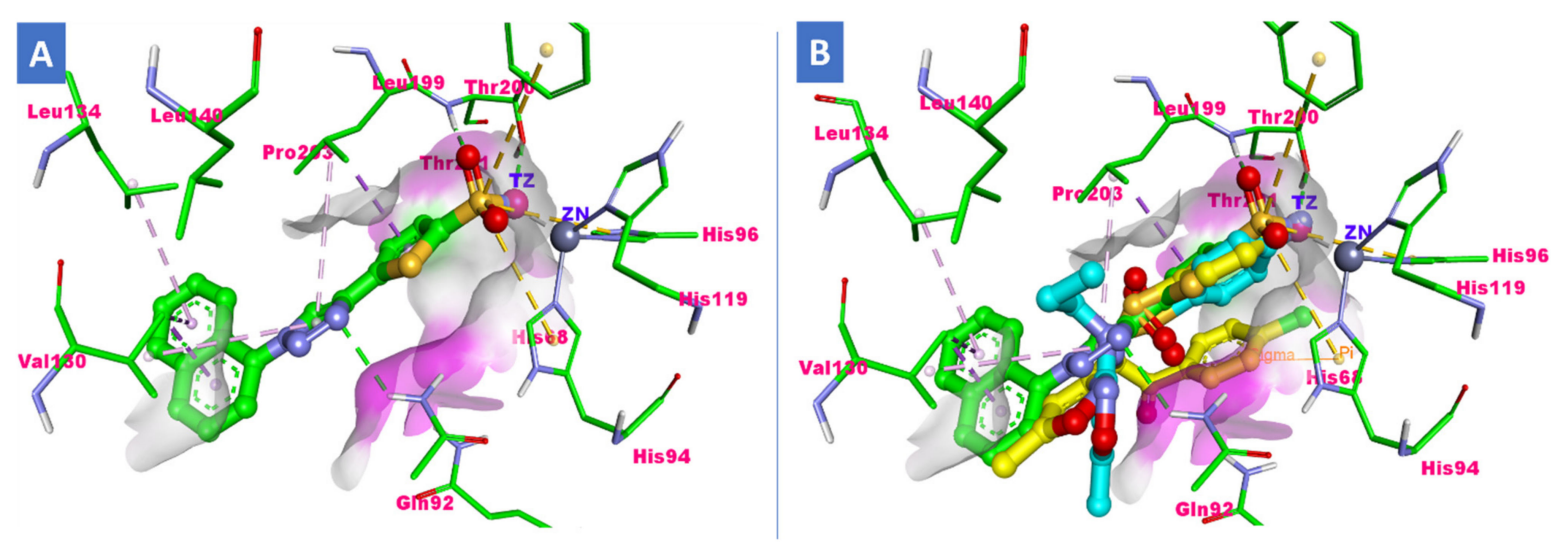
(**A**) 3D binding pattern of 9fk (green) into active place of hCA-IX isoenzyme; (**B**) Overlay of **S3**, **S15** and 9FK to show the shape complementarities with hCA-IX active place and the difference in torsions and conformations. The docked and redocked poses were displayed in ball & stick style. Zn atom was shown as a blue ball while pseudoatom TZ was represented as a pink ball. Favorable co-ordinate bond with Zn cation, hydrogen bonds and π-Stacking were showed in dashed blue, green and orange dashed lines, respectively. Hydrogen bond surfaces around docked poses were created. Hydrogen atoms were omitted for clarity purpose.

**Figure 10:**
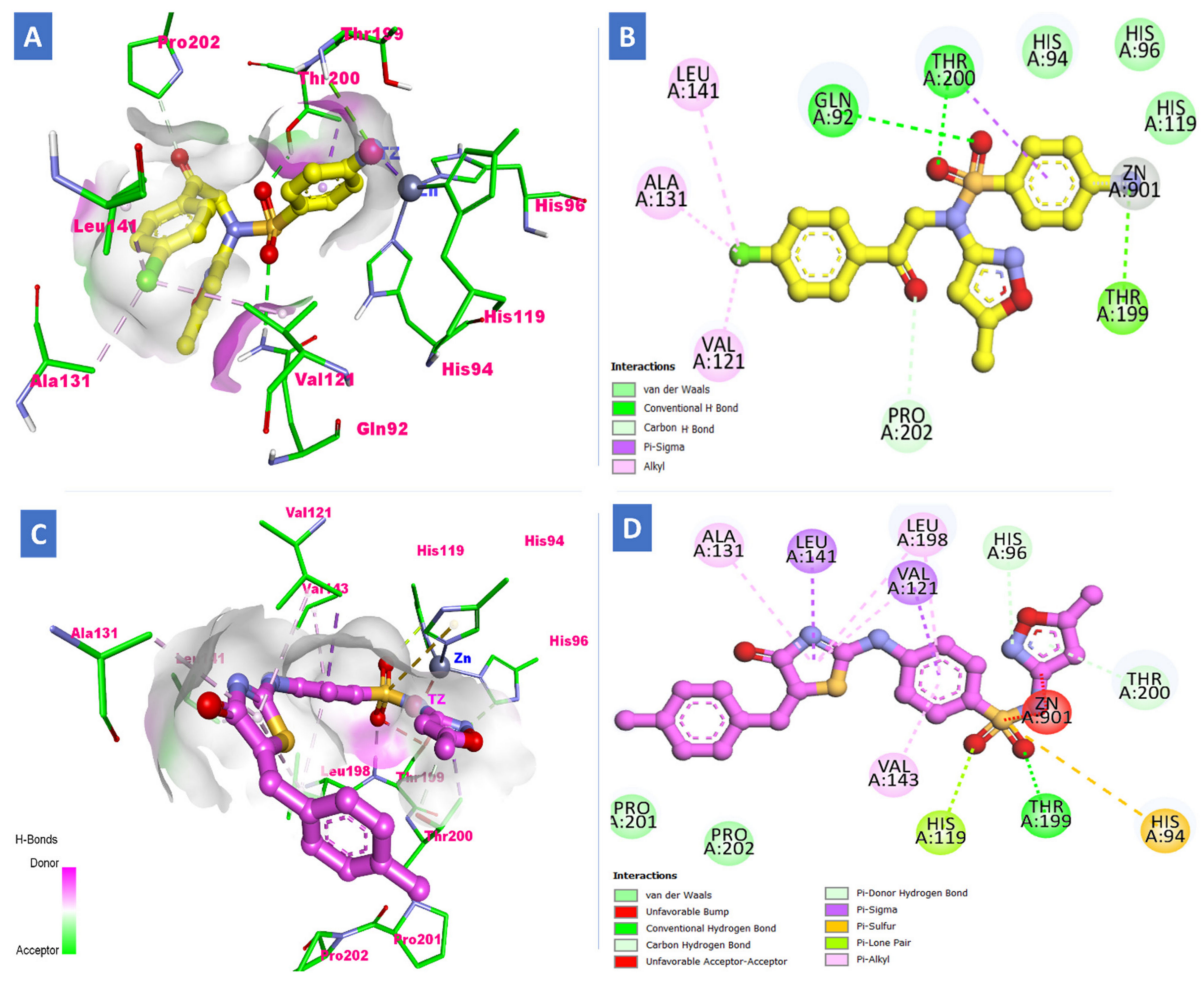
The docking results and interactions of compounds **S15** (yellow) and **S19** (violet) into the active place of hCA-XII isoenzyme (PDB code: 1JD0); (**A**,**B**) 3D and 2D binding modalities of **S15**, respectively; (**C**,**D**) 3D and 2D binding modalities of **S19**, respectively. The docked poses were displayed in ball & stick style. Zn atom was shown as a blue ball while pseudoatom TZ was represented as a pink ball. Unfavorable co-ordinate bond with Zn cation or clashes were displayed in dashed red lines. π -Sulfur and hydrophobic interactions were represented as orange and light violet dashed lines, respectively. Hydrogen bond surfaces around docked poses were created. Hydrogen atoms were omitted for clarity purpose.

This study was achieved using *Autodock Vina program version 1.2.0 0* [42,43]. The details of the docking method, visualization and crystal structure were downloaded from the protein data bank and carried out according to reported methods; the details are presented in Supplementary Material Section S2.3 [43,44] (Table 5, Figure 7, Figure 8, Figure 9 and Figure 10).

## 4. Conclusions

The new synthesized compounds possess carbonic anhydrase I, II, IX and XII inhibitory activities. The activity of compounds **S9** and **S15** towards hCAs may be attributed to 4-chloropheny groups; however, the presence of methyl groups in compounds **S8** and **S18** also showed activity and selectivity, but less than chloride. Additionally, amido groups in the synthesized compounds (**S8–S11**, **S14–S17**) showed a vital role in binding with the zinc atom in receptor (ZBG) and this reflected on its activity.

The sulphomyl moieties possess effects similar to the urido linker. The thiazolidinone rings (**S18–S22**) did not enhance activity as expected, as it was weakly bonded with ZBG.

The strong electron withdrawn group (nitro, **S10**) or donating group (trimethoxy groups; **S11**) had no impact on activity. The **S15** has selective inhibitory activity towards hCAs IX and X11 compared to hCAs I and II, paving the way for the discovery of new selective anticancer agents.

## Figures and Tables

**Table 1:** Inhibitory activity of compounds **S1–S22** against hCAIX, and hCAXII using Dorzolamide HCL as reference standard.

Compound	Carbonic Anhydrase (IC_50_, nM)	
Compound	CA-IX	CA-XII
**S1**	0.119 ± 0.006	0.162 ± 0.008
**S2**	0.083 ± 0.004	0.056 ± 0.003
**S3**	0.042 ± 0.002	0.07 ± 0.003
**S4**	0.172 ± 0.008	0.208 ± 0.01
**S5**	0.398 ± 0.019	0.651 ± 0.032
**S6**	1.664 ± 0.081	0.877 ± 0.043
**S7**	0.413 ± 0.02	0.18 ± 0.009
**S8**	0.042 ± 0.002	0.04 ± 0.002
**S9**	0.074 ± 0.004	0.047 ± 0.002
**S10**	0.789 ± 0.039	0.392 ± 0.019
**S11**	1.977 ± 0.097	0.991 ± 0.048
**S12**	0.902 ± 0.044	0.437 ± 0.021
**S14**	0.152 ± 0.007	0.081 ± 0.004
**S15**	0.037 ± 0.002	0.061 ± 0.003
**S16**	0.165 ± 0.008	0.164 ± 0.008
**S17**	0.235 ± 0.011	0.247 ± 0.012
**S19**	1.587 ± 0.078	0.917 ± 0.045
**S20**	0.51 ± 0.025	0.069 ± 0.003
**S21**	0.698 ± 0.034	0.284 ± 0.014
**S22**	0.186 ± 0.009	0.249 ± 0.012
Dorzolamide HCL	0.036 ± 0.002	0.024 ± 0.001

The data in the table represent the average ± SEM after three independent experiments. and consider statistically significant at *p* < 0.05 if compared to DZM for CA-IX and CA-XII, respectively.

**Table 2:** Human CAI, II inhibitory effect of **S2**, **S3**, **S8**, **S9** and **S15** compounds.

Compound		Carbonic Anhydrase IC_50_ (uM)	
Code	MW g/mol	hCA I	hCA II
**S2**	293	0.177 ± 0.008	0.102 ± 0.006
**s3**	295	0.08 ± 0.003	0.199 ± 0.011
**S8**	471	1.017 ± 0.049 ^a^	1.848 ± 0.12 ^b^
**S9**	391	0.121 ± 0.006	0.543 ± 0.03 ^b^
**S15**	420	0.411 ± 0.02	0.158 ± 0.009
AAZ	222.245	0.281 ± 0.014	0.117 ± 0.007

The data in the table represent the average ± SEM after three independent experiments. ^a^ and ^b^ consider statistically significant at *p* < 0.05 if compared to AAZ for hCAI and hCAII, respectively.

**Table 3:** Effect of **S8**, **S9** and **S15** compounds on cell viability of MCF7 (normoxia and hypoxia) and MCF10a cells using Staurosporine as a reference standard.

Sample		Cytotoxicity (IC_50_ µg/mL)		
Compound	MW	MCF7		MCF10a
		Hypoxia	Normoxia	
**S8**	471	3.64 ± 0.2	7.68 ± 0.41	31.65 ± 1.71
**S9**	391	9.81 ± 0.53	24.69 ± 1.33	46.72 ± 2.52
**S15**	420	0.73 ± 0.04	4.15 ± 0.22	34.71 ± 1.87
**Staurosporine**	466.5	1.63 ± 0.09	4.78 ± 0.26	23.65 ± 1.27

The data in the table represent the average ± SEM after three independent experiments.

**Table 4:** Effect of compound **S15** treatment of MCF7 cancer cell line on necrosis and apoptosis.

	Apoptosis			Necrosis
	Total	Early	Late	
**S15/MCF7**	41.03	2.92	22.8	15.31
**Control/MCF7**	1.35	0.29	0.07	0.99

The data in the table represent the average ± SEM after three independent experiments.

**Table 5:** The collective docking results and the interactions of compounds **S3** and **S15** into *h*-CA-IX isoenzyme and **S15** and **S19** into *h*-CA-XII isoenzyme.

Ligand (Isoenzyme)	ΔG (kcal/mol)	H-Bonding Interactions		Hydrophobic Interactions	π-π Stacking
		Chemical Group	Amino Acids Involved		
**S3** (h-CA-IX)	−29.86	Amino and sulfonamide	Thr200, Gln92	Leu91, Val130 and Leu140	p-chlorophenyl ring with His94
**S15** (h-CA-IX)	−28.98	Amino, isoxazole and sulfonamide	Thr200, Gln92	Leu91, Val130 and Leu140	p-chlorophenyl ring with His94
**S15** (h-CA-XII)	−27.83	Amino and sulfonamide	Thr199, Gln92 and Thr200	Val121, Ala131, Leu141 and Leu198	--
**S19** (h-CA-XII)	−25.21	sulfonamide	Thr199	Val121, Ala131, Leu141 and Val143	--

## Data Availability

Data is contained within the article and Supplementary Material.

## Supplementary Materials

The following supporting information can be downloaded at: https://www.mdpi.com/article/10.3390/ph15091134/s1.
