# Supplementary material for: New Sulfamethoxazole Derivatives as Selective Carbonic Anhydrase IX and XII Inhibitors: Design, Synthesis, Cytotoxic Activity and Molecular Modeling
Source: Pharmaceuticals (Basel). 2022 Sep 10;15(9):1134. doi: 10.3390/ph15091134 (PMC9501356; doi:10.3390/ph15091134)
Supplement: Supplementary file 1 [file pharmaceuticals-15-01134-s001.zip › pharmaceuticals-1864049-supplementary.pdf]

S2. Experimental

S2.1. Chemistry

Uncorrected melting points were assessed using Griffin apparatus . Infrared (IR) spectra were recorded as films on KBr disc using a Nicolet 550 Series II Magna FT-IR spectrometer. ^{1}H NMR and ^{13}C NMR spectra were recorded on Bruker apparatus at 400 MHz for ^{1}H NMR and 100 MHz for ^{13}C NMR (DEPT-Q) spectrophotometer, (Faculty of Pharmacy, Beni-Suef University, Beni Suef, Egypt), using DMSO-d_6 as a solvent and TMS as the internal standard, where *J* (coupling constant) values were estimated in Hertz (Hz) and chemical shifts were recorded in ppm on δ scale. Mass spectra (MS) were determined on Hewlett Packard 5988 spectrometer at the regional center for mycology and biotechnology, Al-Azhar University, Egypt. Microanalyses for C, H, and N were carried out on Perkin-Elmer 2400 analyzer (Perkin,-Elmer, Norwalk, CT, USA) at the regional center for mycology and biotechnology, Al-Azhar University, Egypt, and all compounds were within ± 0.4% of the theoretical values. Progress of the reactions was monitored using thin-layer chromatography (TLC) sheets that were precoated with UV fluorescent silica gel MERCK 60 F 254 that was visualized by the UV lamp. The solvent system was chloroform: methanol (in different ratios). Sulphamethexazole and all other chemicals purchased from the Aldrich Chemical Company (Milwaukee, WI), were used without further purification. Compounds **S5, S13a-d, a**nd **S18** were prepared according to a reported procedures.

Instruments specifics used for NMR , IR, mass spectroscopy and elemental analysis of synthesis compounds are presented as supplementary data (supp-2). Also TLC plates and chemical supply are illustrated in supp-2

S2.2. Biological activities

S2.2.1. Materials and methods

S2.2.1.1. Chemicals and kits

Annexin V-FITC Apoptosis Detection Kit was purchased from Biovision Research Products (CA, USA). Recombinant human carbonic anhydrase I and recombinant human carbonic anhydrase XII were purchased from R and D system (MN, USA). Recombinant human carbonic anhydrase II and recombinant human carbonic anhydrase IX were purchased from BPS bioscience (CA, USA). Insulin for culture media and *In vitro* toxicology assay kit (MTT based) were obtained from Sigma-Aldrich (MI, USA). Cell culture media DMEM was obtained from Invitrogen/Life Technologies (CA, USA). Fetal bovine serum was purchased from Hyclone (UT, USA).

S2.2.2. CAIX inhibitory assay

The hCAIX was assayed following the manufacturer's instruction. In brief, 1,400 pmol/min/µg reaction mixture of 15 mM Tris-HCl, pH 7.4, 100mM NaCl, and 3mM p-Nitrophenyl acetate (PNPA). A 96-well plates containing 100 ng of enzyme diluted with buffer, water with final concentration of 1mM PNPA and the selected compound and concentration to be tested. The

mixture was incubated for 30 min at room temperature and the absorbance was measured at 405nM. [39]

S2.2.3. hCAXII inhibitory assay

Carbonic Anhydrase (CA) catalyzes the reversible reaction of CO2 + H2O = $HCO3^-$ + H^+. Carbonic Anhydrase I (CAI) was measured following the manufacturer's instruction. In brief, recombinant Human Carbonic Anhydrase XII (rh CAXII) was diluted to 2 mM in assay buffer (12.5 mM Tris, 75 mM NaCl, pH 7.5). The substrate (4-Nitrophenyl acetate, 4NPA, 100 mM stock in acetone) was diluted in 2 mM in assay buffer. The reaction was started by adding 50 μL of 2 mM substrate with 50 μL of 20 ng/μL rhCAXII in 96 well plate. Substrate blank containing 50 μL of assay buffer and 50 μL of substrate was included. The kinetics for 5 min of the reaction mixtures were measured at wavelength of 400 nm[40] and the activity was calculated using the following equation:

$$\text{Specific Activity (pmol/min/}\mu\text{g)} = \frac{\text{Adjusted } V_{max} \text{ (OD/min) x Conversion Factor (pmol/OD)}}{\text{amount of enzyme (}\mu\text{g)}}$$

S2.2.4. CA1 inhibitory assay

Carbonic Anhydrase (CA) catalyzes the reversible reaction of CO_2 + H_2O = HCO_3^- + H^+. Carbonic Anhydrase I (CAI) was measured following the manufacturer's instruction. In brief, recombinant Human Carbonic Anhydrase I (rh CAI) was diluted to 2 mM in assay buffer (12.5 mM Tris, 75 mM NaCl, pH 7.5). The substrate (4-Nitrophenyl acetate, 4NPA, 100 mM stock in acetone) was diluted in 2 mM in assay buffer. The reaction was started by adding 50 μL of 2 mM substrate with 50 μL of 100 ng/μL rhCAI in 96 well plate. Substrate blank containing 50 μL of assay buffer and 50 μL of Substrate was included. The kinetics for 5 min of the reaction mixtures were measured at wavelength of 400 nm[41] and the activity was calculated using the following equation:

$$\text{Specific Activity (pmol/min/}\mu\text{g)} = \frac{\text{Adjusted } V_{max}\text{* (OD/min) x Conversion Factor** (pmol/OD)}}{\text{amount of enzyme (}\mu\text{g)}}$$

S2.2.5. CAII inhibitory assay

CAII was assayed following the manufacturer's instruction. A amount of 1,400 pmol/min/μg assay mixture containing 100 mM NaCl, 15 mM Tris-HCl, pH 7.4, 3 mM p-Nitrophenyl acetate (PNPA) and 0-2 μg CAII. Incubate for 30 min at RT and the resulted absorbance was measured at 405 nm. [42]

S2.2.6. Cell culture Protocol

MCF7 and MCF10a cell lines were obtained from American Type Culture Collection, the cultured cells were grown using DMEM (Invitrogen/Life Technologies) in 10% fetal bovine serum (FBS), 10 μg/ml of insulin (Sigma), and 1% penicillin-streptomycin antibiotics. Other chemicals and reagents were from obtained from Sigma or Invitrogen. Cells density was 1.2 – 1.8 × 10^3 cells/well in a volume of 100 μl complete growth medium + 100 μl of the tested compound/well in a 96-well plate for 24 hours performing

assay. After treatment of cells with the serial concentrations of the tested compounds, incubation for 48 h at 37ºC is carried out.

S2.2.7. MTT – Cytotoxicity assay protocol

MTT assay is a method used for *in vitro* cytotoxicity of different compounds. MTT vial was reconstituted in 3 ml growth media and diluted MTT in an amount equal to 10% of the culture medium volume. After 4hrs incubation of the reagent with the treated MCF7 and MCF10a cells, culture media was removed from incubator and dissolve the resulting formazan crystals by adding an amount of MTT solubilizing solution equal volume with the original culture medium. Absorbance was measured spectrophotometrically at 570 nm wavelength using microplate reader (Bio-Rad, Molecular Devices, CA, USA).

S2.2.8. Annexin V-FITC Assay for apoptosis

Treated MCF7 and MCF10a cells of 1-5 x 105 were collected and suspended with binding buffer and cultured with incubated with 5 µl Annexin V-FITC and 5 µl of propidium iodide (PI 50mg/ml). Cells were incubated at room temperature for 5 min in the dark. Annexin V-FITC binding analysis is done by flow cytometry (Ex = 488 nm; Em = 530 nm) using FITC signal detector (FL1) and PI staining by the phycoerythrin emission signal detector (FL2).

S2.3. Docking Study

This study was performed employing *Autodock Vina program version 1.2.0 0.* [36]–[38]. 3D Crystal structures of *h*CA-IX enzyme (PDB ID: 5FL4) and *h*CA-XII (PDB code: 1JD0) were selected and downloaded from Protein Data Bank (http://www.rcsb.org). The co-crystallized ligands were extracted from the initial crystal structures followed by deleting all water molecules. the docked compounds were energy minimized and rotatable bonds were adjusted and then, they were saved as PDBQT files. The Swiss-PdbViewer software version 4.1.0 was used to prepare both proteins by adding the missing atoms and energy minimization. Then, the polar hydrogens and Gastieger charges were added, and the corresponding charge files were generated, and the structures were saved in PDBQT file format using the *Autodock Tools (ADT) version 1.5.6* according to the previous reports[43] . Subsequently, a tetrahedral Pseudo Zinc atom (*TZ*) was added to the protein using python embedded with AutoDock Vina. Thereafter, the affinity maps were generated and a special GPF file for docking with Pseudo Zn atoms was obtained. At this stage, the autogrid was performed and then, the docking process started using the specialized *AutoDock4Zn* forcefield. The exhaustiveness was adjusted to 32. The settings of the two grid boxes were selected to encompass the co-crystallized inhibitors inside the active sites of both targets. The center co-ordinate was obtained from the central atom of the co-crystallized ligand. The *h*CA-IX box was centered toward the coordinates of (X = 14.263, Y = -26.674, Z = 59.107 Å) with size of (X= 40, Y = 20, Z = 20 Å), while *h*CA-XII box was centered toward the coordinates of (X= 18.48, Y= 6.52, Z = 25.56 Å) with size of (X= 20, Y = 30, Z = 20 Å). The results including the 2D and 3D binding patterns were visualized using *Discovery Studio Visualizer* (DSV), V21.1.0.20298 (*BioVia*, San Diego, CA, USA) [44].
